# *Gentiana tingnongiana* and *G. shangwui*, two new species of *Gentiana* (Gentianaceae) from the eastern Qinghai-Tibet Plateau, Sichuan province, China

**DOI:** 10.3897/phytokeys.275.190176

**Published:** 2026-05-27

**Authors:** Hai-Feng Cao, Liang-Hong Ni, Jie Cai, Ji-Dong Ya, Gan Li, Yue-Qing Luo, Peng-Cheng Fu, Lei Zhang

**Affiliations:** 1 Shanghai Museum of TCM, Shanghai University of Traditional Chinese Medicine, Shanghai, 201203, China Shanghai Museum of TCM, Shanghai University of Traditional Chinese Medicine Shanghai China; 2 Shanghai University of Traditional Chinese Medicine, Shanghai, 201203, China Shanghai University of Traditional Chinese Medicine Shanghai China; 3 Germplasm Bank of Wild Species, Kunming Institute of Botany, Chinese Academy of Sciences, Kunming, Yunnan, 650201, China School of Life Science, Luoyang Normal University Luoyang China; 4 School of Life Science, Luoyang Normal University, Luoyang, 471934, China Kunming Institute of Botany, Chinese Academy of Sciences Kunming China; 5 School of Biological Science & Engineering, North Minzu University, Yinchuan, 750021, China School of Biological Science & Engineering, North Minzu University Yinchuan China

**Keywords:** China, *Gentiana*, Gentianaceae, new species, Sichuan Province

## Abstract

*
Gentiana tingnongiana* H.F.Cao & L.Zhang, **sp. nov**., and *Gentiana shangwui* H.F.Cao & L.Zhang, **sp. nov**., two new species of Gentianaceae from Sichuan, China, are described and illustrated here. *G. tingnongiana* has an erect main stem, obovate to oblong stem leaves, a yellowish-green corolla with external stripes and a pink to pale purple or sub-white interior, numerous blackish-purple spots in the throat and plicae, and small seeds 0.7–1.0 mm in diameter. *Gentiana shangwui* is characterized by densely puberulent leaves and calyces, a corolla throat without short striations, a bright yellow or pale yellow middle part of the corolla tube, a relatively small corolla, stamens inserted at the apical part of the tube, and a 4–6 mm long style. Phylogenetic analysis of internal transcribed spacer sequences recovers both species as distinct, well-supported monophyletic lineages. Combined morphological and phylogenetic evidence supports the establishment of these two new species.

## Introduction

*Gentiana* Tourn. ex L. is the most species-rich genus in the Gentianaceae family and currently contains approximately 450 species (Ho and Pringle 1995; Ho and Liu 2001; Ho et al. 2002). It occurs in mountain systems worldwide and is most diverse in temperate and montane tropical regions (Ho and Liu 2001; Favre et al. 2016; Sun et al. 2026). Approximately 250 *Gentiana* species occur in China. Most of them have a narrow distribution range and exhibit distinct regional characteristics, with nearly half being endemic (Struwe and Pringle 2018; Chen et al. 2021; Chen et al. 2024). Members of the genus share a set of distinct common morphological features, most notably the presence of glands at the base of the ovary and folds between the corolla lobes, both of which hold significant taxonomic identification value (Cao et al. 2019; Yang et al. 2020; Favre et al. 2022; Sun et al. 2026).

The western plateau of the Sichuan Province is a key component of a global biodiversity hotspot. Located on the eastern edge of the Qinghai-Xizang Plateau, this region has a complex landscape and significant altitude differences. It provides a crucial refuge and unique ecological niche for plants and is home to a large number of species such as *Rhododendron* L., *Pedicularis* L., and *Gentiana* (Favre et al. 2016). In 2014, we discovered two small populations of *Gentiana* in the Balang Mountains and Songpan. Initial morphological observations indicated that these plants could not be assigned to any known species; therefore, it was speculated that they might be new species (Suppl. material 1: figs S1, S2). To clarify their taxonomic status, we conducted detailed morphological comparisons and obtained molecular evidence to confirm their identity and to provide new data for the classification and systematic evolution of *Gentiana*.

## Material and methods

### Morphological analyses

**Figure 1.**
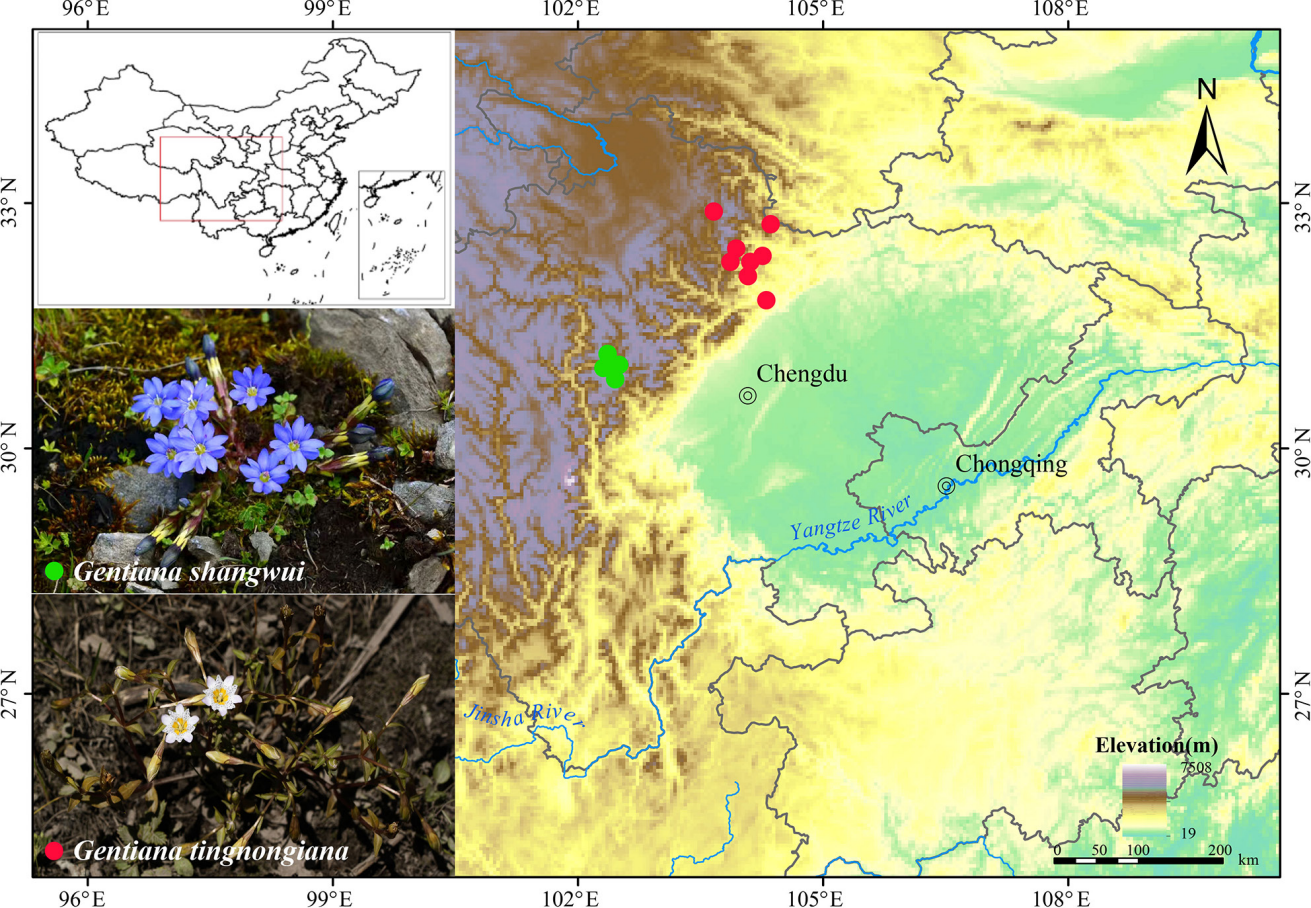
Distribution map of *G. tingnongiana* and *G. shangwui*.

Herbarium specimens and silica gel-dried leaves of *Gentiana tingnongiana* and *G. shangwui* were collected from Songpan County and the Balang Mountains in western Sichuan Province, Southwest China (Fig. 1). Type specimens of the two new species were deposited in the herbaria CSH, KUN, LYUH, PE, and SMCM. The measurements and descriptions of the morphological characteristics of the new species were based on dried specimens and living plants. High resolution specimen pictures of *G. filisepala* T.N.Ho, *G. baoxingensis* T.N.Ho, *G. vandellioides* Hemsl., *G. rubicunda* Franch., *G. piasezkii* Maxim., *G. winchuanensis* T.N.Ho, and *G. nanobella* C.Marquand were obtained online (including CVH, GBIF, JSTOR, etc.), and specimens deposited in CDBI, CQNM, HNWP, IBSC, KUN, PE SM and SZ herbaria were carefully checked. An average of two or three mature leaves, flowers, and fruits were chosen to conduct measurements for each of the specimens using ImageJ version v1.53t (Schneider et al. 2012). Final morphometric analyses were conducted on leaf, flower, and fruit traits for a number of species that are morphologically close to the two new species. The variability in each of the six quantitative traits studied in detail is shown in boxplots generated using R software (Team 2017). Analysis of variance (ANOVA) was performed for each quantitative trait using SPSS version 19 (IBM 2010). Subsequently, principal component analysis (PCA) was conducted using the R package ggrepel version 0.9.5 (https://ggrepel.slowkow.com) and visualized using the ggplot2 package (Wickham 2011). Suppl. material 1: tables S1, S2 give the list of specimens studied for the species which were compared with the new species. The line drawings, the descriptions and most photographs were based on type specimens. The Extent of Occurrence (EOO) was calculated using ArcGIS. First, the occurrence points of the species (including both collected samples and herbarium specimens) were projected in the software, and then the area of the resulting polygon was calculated. The conservation status of the new species was evaluated according to the International Union for Conservation of Nature (IUCN) Red List Categories and Criteria (IUCN 2024).

### ITS sequences and phylogenetic analyses

The total genomic DNA was extracted from the dried leaves using the hexadecyl trimethyl ammonium bromide (CTAB) method (Doyle and Doyle 1987). The regions of partial internal transcribed spacer 1, and partial internal transcribed spacer 4 were amplified using previously reported ITS1 (5’-GGAAGTAGAAGTCGTAACAAGG-3’) and ITS4 (5’-TCCTCCGCTTATTGATATGC-3’) primers (White et al. 1990). Polymerase chain reactions (PCRs) were performed using a 25 μL reaction mix comprising 2.5 μL of 10× Taq buffer, 0.5 mM of each dNTP, 50–100 ng of diluted genomic DNA, 0.5 μL of each primer, and 0.5 units of Taq polymerase (Vazyme Biotech, Nanjing, China). The cycling conditions were as follows: a single cycle at 94 °C for 4 min, followed by 36 cycles of 94 °C for 50 s, 53 °C for 50 s, and 72 °C for 50 s, with a final extension cycle at 72 °C for 10 min (Favre et al. 2016). The amplified products were analyzed using an ABI 3830xl DNA analyzer (Applied Biosystems Inc., Foster City, CA, USA) at Nuohe Biological Technology (Tianjin, China). To establish the phylogenetic placement of *G. tingnongiana* and *G. shangwui*, we reconstructed an internal transcribed spacer (ITS) phylogenetic tree using 79 sequences representing 71 species, including 76 *Gentiana* sequences (from 68 species) and three outgroups. Of these, 67 sequences were downloaded from the NCBI database, and the remaining 12 were obtained from our laboratory (Suppl. material 1: table S3).

Multiple sequence alignment was performed using MEGA 7.0 (Kumar et al. 2016) with ClusterW and was subsequently manually adjusted. Maximum Likelihood (ML) phylogenetic reconstruction was conducted in RAxML version 8.1.24 software (Stamatakis 2014) and run by Iqtree 2.0.3 (Nguyen et al. 2014) with 1000 bootstraps. The final phylogenetic trees were visualized using FigTree version1.4.4 (Rambaut 2018).

## Results

### Morphology

Character analysis showed differences between *G. tingnongiana* H.F.Cao & L.Zhang, *G. filisepala* T.N.Ho, *G. baoxingensis* T.N.Ho, *G. vandellioides* Hemsl., and *G. rubicunda* Franch. in the following characters (Table 1), color and structure of main stem, shape and size of leaves, size of calyx lobes and pubescence of their margins, corolla length and colo, plicae, insertion of stamens and lengths of filaments, seed size and flowering period.

**Table 1.** Morphological comparisons between *G. tingnongiana*, *G. filisepala*, *G. baoxingensis*, *G. vandellioides* and *G. rubicunda*.

	* G. tingnongiana*	* G. filisepala*	* G. baoxingensis*	* G. vandellioides*	* G. rubicunda*
Plant height	5–22 cm	10–20 cm	3–4 cm	6–10 cm	8–15 cm
Stems	purplish-red, pale brown or green, erect with a main stem, sometimes ascending	purple, ascending, much branched from base	purple, ascending, little branched from base	yellowish-green, ascending, dichotomously branched from base	purple or green, erect with a main stem, simple or little branched from middle
Stem leaves	8–15 × 4–11 mm, lower stem leaves obovate or obovate-elliptic, upper stem leaves oblong, margin ciliolate	5–8 × 3–3.5 mm, all stem leaves ovate, margin almost smooth	2.5–3.5 × 2–3 mm, all stem leaves ovate, margin hirsute	6.5–12 × 3.5–6 mm, all stem leaves broadly ovate to subcordate, margin smooth or ciliolate	4–22 × 2–7 mm, lower stem leaves ovate-elliptic, ovate, or obovate-orbicular, upper stem leaves oblong, margin ciliolate
Calyx lobes	2.0–3.5 mm long, margin smooth	3.5–4.5 mm long, margin smooth	1.5–2 mm long, margin hirsute	2–2.5 mm long, margin smooth or ciliolate	3–6 mm long, margin smooth
Corolla color	pink, pale purple or subwhite, throat with blackish-purple spots	purple, throat with numerous blackish short lines	purple, throat with numerous blackish short lines	pale blue, throat without stripe and spots	purple, throat with numerous dark purple short lines and spots
Corolla length	0.9–1.5 cm long	(1.2–) 1.7–2 cm long,	0.9–1.0 cm long	1.2–1.5 cm long	1.5–6 cm long
Plicae	triangular, regular, margin entire, with blackish-purple spots	broadly oblong, margin erose, without spots	triangular to broadly ovate, margin entire or denticulate, without spots	ovate, margin entire or 2-cleft, without spots	ovate, margin entire, erose or 2-cleft, without spots
Stamens	inserted slightly below middle of corolla tube, filaments 4–5 mm long	inserted at middle of corolla tube, filaments 3.5–4 mm long	Inserted just above middle of corolla tube, filaments1–1.2 mm long	inserted just below middle of corolla tube, filaments 4–5 mm long	inserted at middle of corolla tube, filaments 7–15 mm long
Seeds	diameter 0.7–1.0 mm	diameter 1.1–1.3 mm	-	diameter 1.3–1.5 mm	diameter 1.0–1.3 mm
Fl. and Fr.	April to July	June to August	August	July to September	March to October

Character analysis showed differences between *G. shangwui* H.F.Cao & L.Zhang, *G. piasezkii* Maxim., *G. winchuanensis* T.N.Ho, and *G. nanobella* C.Marquand in the following characters (Table 2): shape and size of basal and stem leaves, corolla length and color, length of corolla lobes and style, stamen insertion and filament length, size and shape of capsules and seeds. *Gentiana shangwui* stands out by having pubescent leaves and calyces, a corolla throat without short striations, an orange-yellow lower corolla tube, a shorter corolla, a distinctly long style (4–6 mm), and apically winged capsules.

**Table 2.** Morphological comparison among *G. shangwui*, *G. nanobella*, *G. piasezkii* and *G. winchuanensis*.

	* Gentiana shangwui*	* G. nanobella*	* G. winchuanensis*	* G. piasezkii*
Basal leaves	suborbicular, 3–6.5 × 2–3 mm	suborbicular, 5–8 × 3–4 mm	ovate, 4–6 × 2–4 mm	involucriform, 17–20 × 4–6 mm
Stem leaves	suborbicular, 2–4 × 2–3.5 mm, both surfaces puberulent and glabrescent	suborbicular, 5–8 × 3–4 mm, both surfaces glabrescent	ovate, 4–6 × 2–4 mm, both surfaces glabrescent	lanceolate, narrowly elliptic, or rarely ovate, 5–9 × 2–3 mm, both surfaces glabrescent
Corolla length	15–20 mm long	20–30(–40) mm long	25–30 mm long	17–22 mm long
Corolla color	dark blue to blue-purple; base yellow-white; throat sparsely spotted or spots absent; outside of corolla tube yellowish with blue-black streaks	dark blue to blue-purple; base sometimes greenish yellow; throat inside with short blackish stripes; outside tinged blackish	blue-purple; throat with yellow and blackish spots	blue-purple to purple; base pale yellow-green; throat with blackish short lines
Corolla lobes	2.5–3.3 mm long	3–5 mm long	6–7 mm long	4–5 mm long
Stamens	inserted at apical part of corolla tube, filaments 4–5 mm long	inserted at middle to apical part of corolla tube, filaments 5.5–6.5 mm long	inserted at middle of corolla tube, filaments 4.5–5 mm long	inserted at or above middle of corolla tube, filaments 4–5 mm long
Style	4–6 mm long	1.2–2 mm long	1–1.5 mm long	2–4 mm long
Capsules	narrowly obovoid to narrowly ellipsoid, 6–6.5 mm long, conspicuously narrowly winged along sutures at apex	cylindric to narrowly ellipsoid, 10–12 mm long, not winged	narrowly ellipsoid, ca 10 mm long, apex long attenuate	narrowly obovoid, 6–8 mm long, conspicuously narrowly winged along sutures at apex
Seeds	1.0–1.5 mm long, coat minutely and densely reticulate	1–1.2 mm long, coat with several irregular spongy ridges	1–1.2 mm long, coat minutely and densely reticulate	1–1.2 mm long, coat minutely and densely reticulate

**Figure 2.**
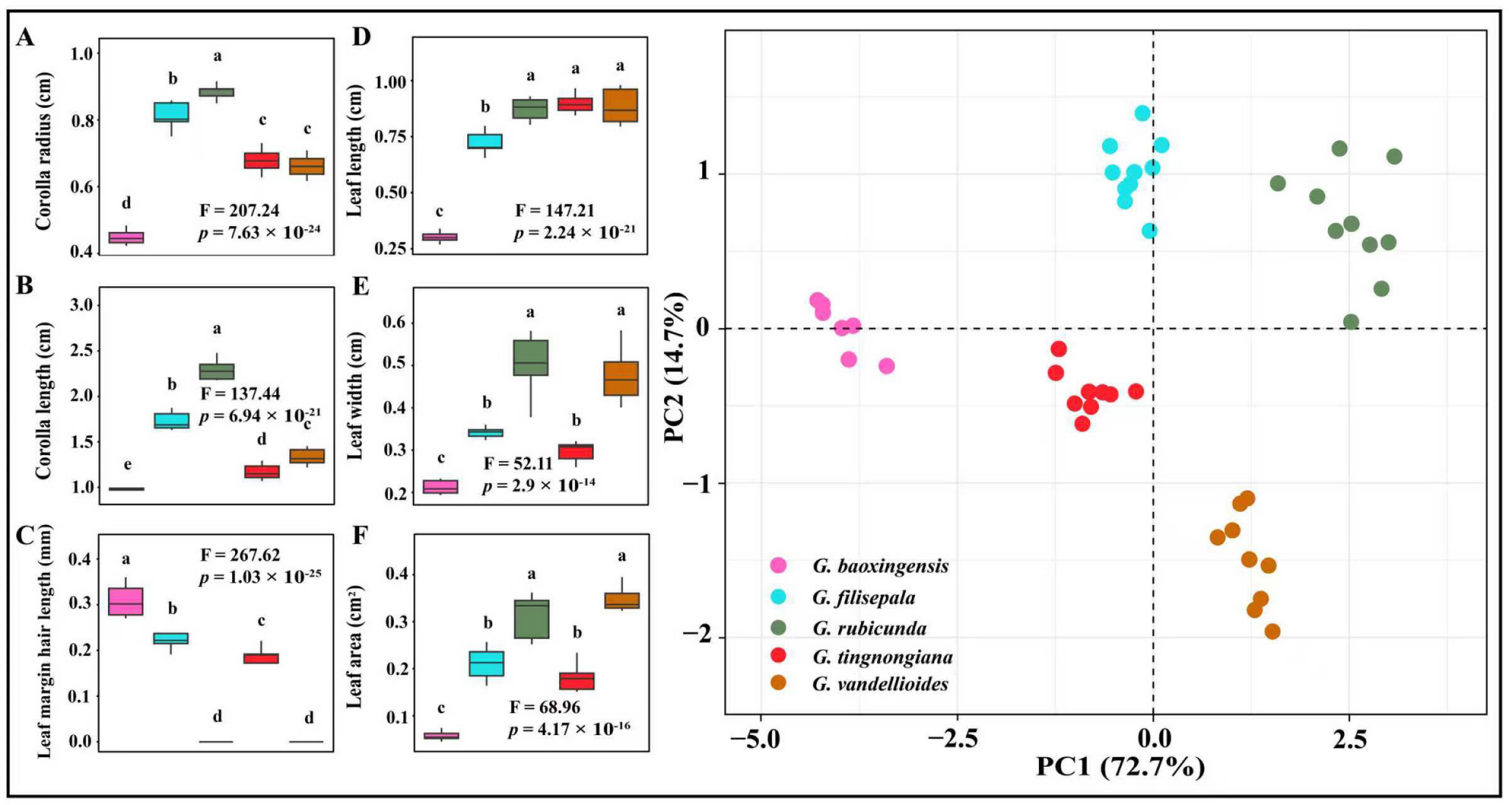
Trait analysis for *G. tingnongiana*, *G. filisepala*, *G. baoxingensis*, *G. vandellioides* and *G. rubicunda*. **A–F**. Results of ANOVA analysis for single trait variation (**A**. Corolla radius; **B**. Corolla length; **C**. Leaf margin hair length; **D**. Leaf length; **E**. Leaf width; **F**. Leaf area. Letters above each of box plots indicate signifcant diferences); **G**. PCA result of six trait variation.

**Figure 3.**
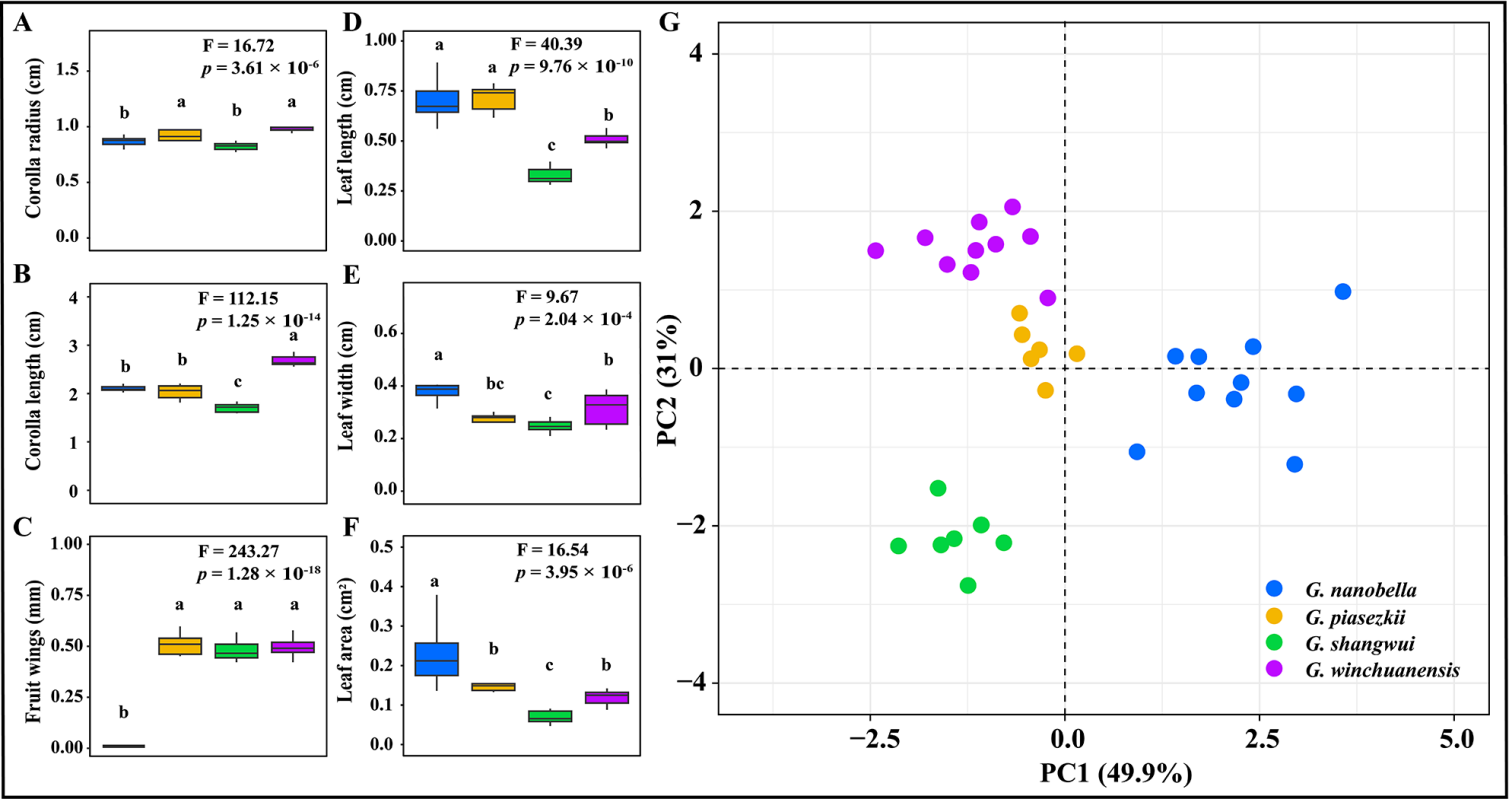
Trait analysis for *G. shangwui*, *G. nanobella*, *G. piasezkii* and *G. winchuanensis*. **A–F**. Results of ANOVA analysis for single trait variation (**A**. Corolla radius; **B**. Corolla length; **C**. Fruit wings; **D**. Leaf length; **E**. Leaf width; **F**. Leaf area. Letters above each of box plots indicate significant differences); **G**. PCA result of six trait variation.

We performed an analysis of variance (ANOVA) on six traits measured of *G. tingnongiana*, *G. filisepala*, *G. baoxingensis*, *G. vandellioides* and *G. rubicunda*: leaf length, width and area, leaf margin hair length, corolla radius and length (Suppl. material 1: table S4, Fig. 2G). We found significant differences in all six traits. Subsequently, we conducted principal component analysis (PCA) on these traits (Suppl. material 1: table S5; Fig. 2A–F). PC1 and PC2 accounted for the majority of shape variation, and the scatter plot of PC1 versus PC2 clearly separated the five species (Fig. 2G). We performed an ANOVA on six traits measured in *G. shangwui*, *G. nanobella*, *G. piasezkii*, and *G. winchuanensis*: leaf length, width and area, corolla radius and length and fruit wings (Suppl. material 1: table S6), and found significant differences in all six traits. Subsequently, we conducted PCA on these traits (Suppl. material 1: table S7; Fig. 3A–F). PC1 and PC2 accounted for the majority of shape variation, and the scatter plot of PC1 versus PC2 clearly separated the four species (Fig. 3G).

For an illustration of the characters of the species *G. tingnongiana* and *G. shangwui* are compared with, see Suppl. material 1: figs S1, S2.

### Molecular analysis

**Figure 4.**
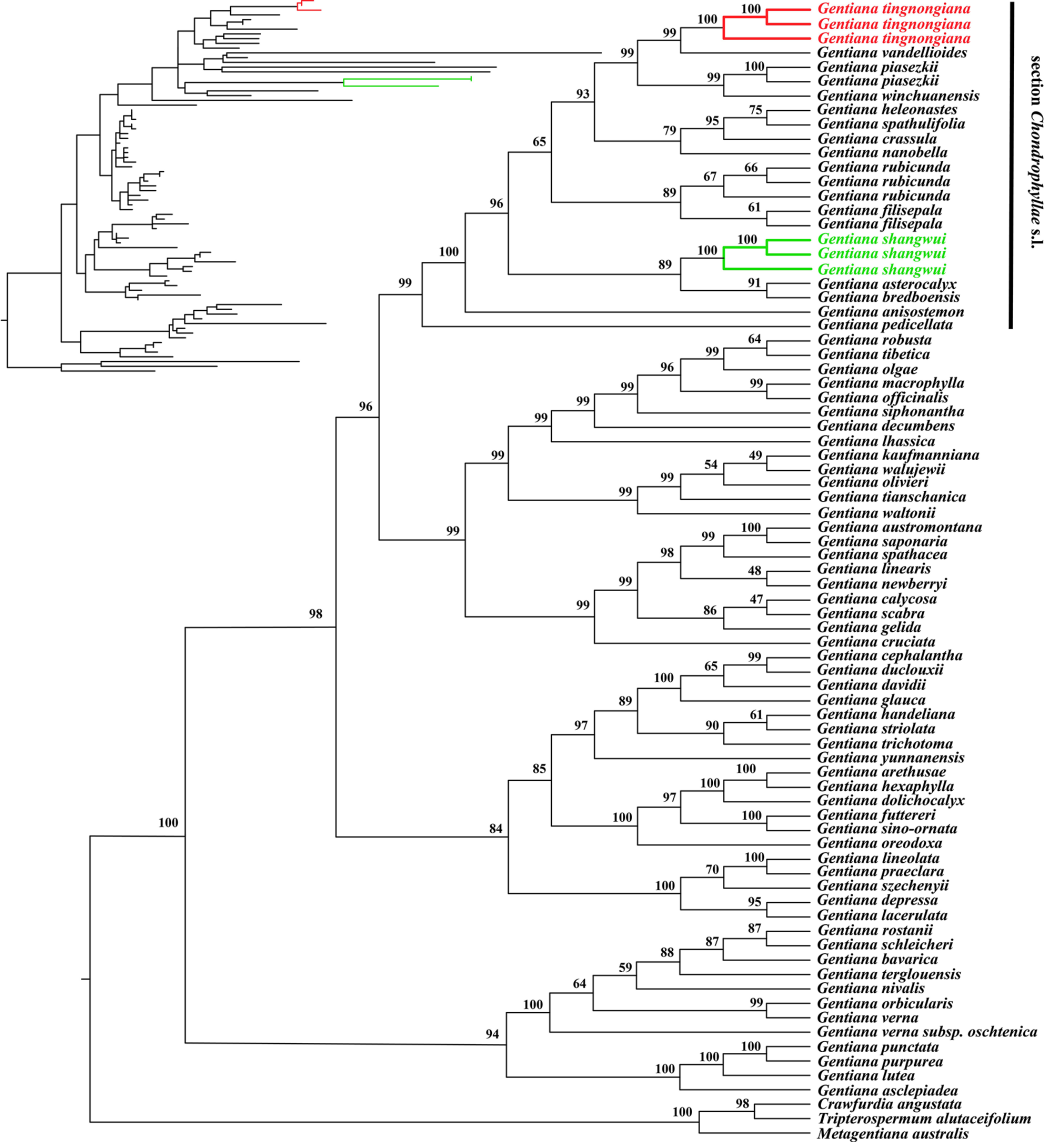
Maximum likelihood phylogenetic tree of 68 *Gentiana* and three outgroup taxa based on ITS. The two inferred new species are highlighted in red and green.

The alignment length of ITS sequences was 630 bp, including 462 variable sites. Phylogenetic analyses revealed that both *G. tingnongiana* and *G. shangwui* were recovered as monophyletic with strong support (Fig. 4; BS = 100%). *Gentiana tingnongiana* was recovered as sister to *G. vandellioides* (BS = 99%). Meanwhile, *G. shangwui* clustered with *G. asterocalyx* Diels and *G. bredboensis* L.G.Adams, also forming a distinct sister relationship (BS = 94%). These two species, along with *G. piasezkii*, *G. winchuanensis*, *G. heleonastes* Harry Sm., *G. spathulfolia* Maxim. ex Kusn., *G. crassula* Harry Sm., *G. nanobella*, *G. rubicunda*, *G. filisepala*, *G. asterocalyx*, *G. bredboensis*, *G. anisostemon* C.Marquand, and *G. pedicellata* (Wall. ex D. Don) Griseb. constituted the monophyletic section *Chondrophyllae* s.l. (BS = 94%).

## Discussion

The description of species is of paramount importance, as these evolutionary units are intricately linked to efforts toward biodiversity conservation and sustainable use (Hamilton 2005; Carstens et al. 2013; Cervantes et al. 2023). However, there is still no scientific consensus on how a species should be defined, an issue that remains highly debated (Wheeler and Meier 2000; Aldhebiani 2017). Under the integrative species concept, species delimitation draws on multiple lines of evidence (Liu 2015, 2016; Sukumaran and Knowles 2017). In botany, evolutionary units that satisfy several diagnostic criteria or conform to multiple species concepts are considered objective, operational, and unbiased taxonomic species (Hu et al. 2015; Zhang et al. 2025).

In this study, we present two independent lines of evidence to support the notion that the two *Gentiana* populations found in Western Sichuan should be recognized as new species. First, individuals from both populations showed morphological differences from all closely related species. Second, previous studies have shown that identifying ITS variations is highly effective for species-level barcoding and resolving taxonomic relationships with *Gentiana* (Chen et al. 2005; Chen et al. 2024). Consistent with this, our phylogenetic analysis based on ITS sequence variations indicated that the individuals of each new species form their own well-supported monophyletic lineage. In addition, both species maintain relatively large populations at the sampling site and could flower and bear fruit normally. This observation indicates that they represent stable and independent evolutionary units. According to the classification criteria of the IUCN, *G. shangwui* is classified as Endangered (EN B1abi), and *G. tingnongiana* as Vulnerable (VU B1abi).

In summary, *G. tingnongiana* closely resembles *G. filisepala*, *G. baoxingensis*, *G. vandellioides* and *G. rubicunda*, and *G. shangwui* is similar to *G. piasezkii*, *G. winchuanensis* and *G. nanobella*, yet each differs from its closest relatives in several consistent morphological traits. Molecular evidence further supports *G. tingnongiana* and *G. shangwui* as distinct monophyletic groups. Together these findings confirm that *G. tingnongiana* and *G. shangwui* represent new species within the genus *Gentiana*. Further molecular and morphological studies will help clarify the taxonomic status and evolutionary relationships of these two new species.

### Taxonomic treatment

#### Gentiana tingnongiana

Taxon classificationPlantaeGentianalesGentianaceae

H.F.Cao & L.Zhang sp. nov.

9E9E820E-5B00-519A-9343-8C0DCA9FFDF5

urn:lsid:ipni.org:names:77380712-1

**Figure 5.**
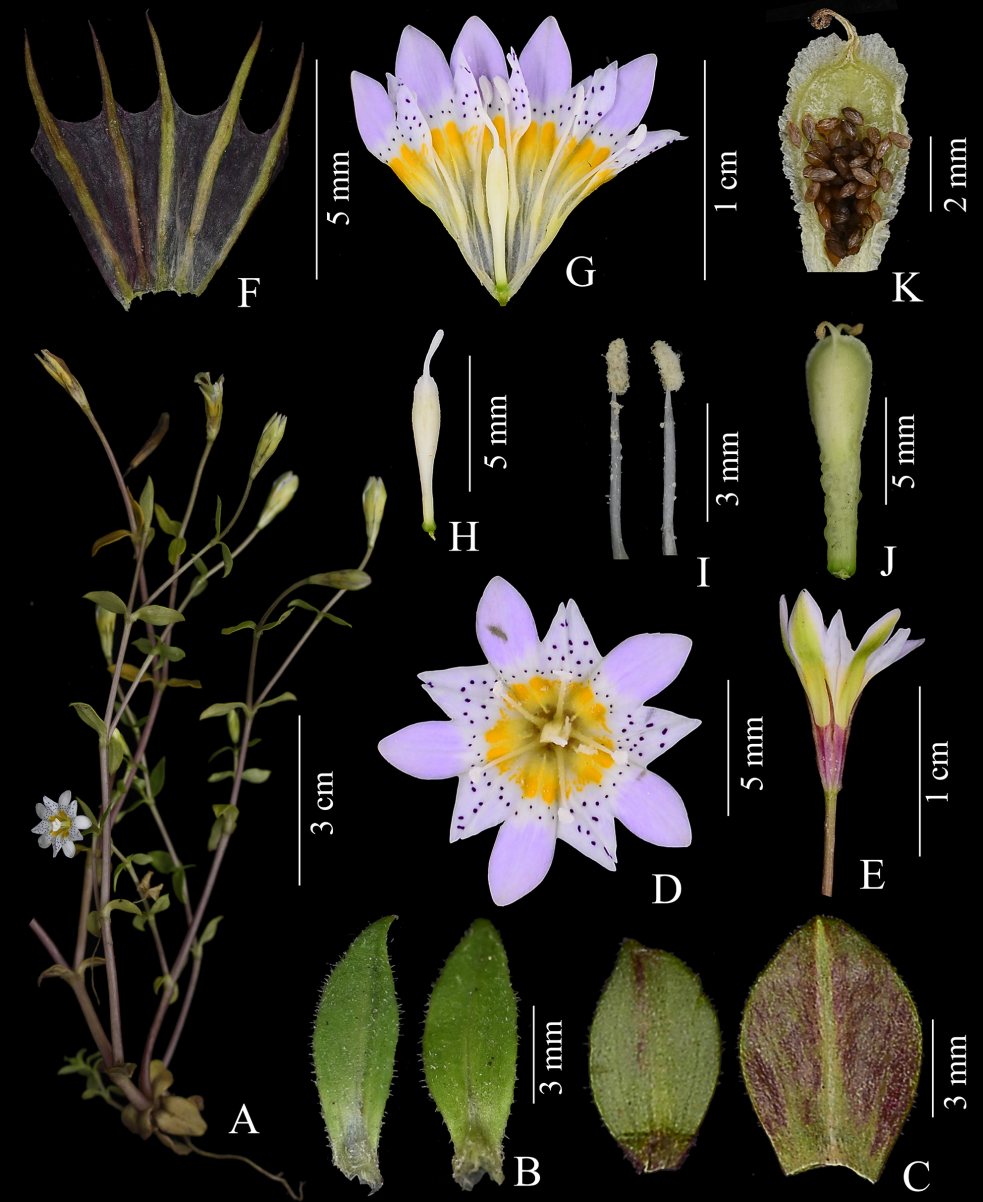
*
Gentiana tingnongiana* H.F.Cao & L.Zhang. **A**. Plant; **B**. Adaxial leaf surfaces; **C**. Abaxial leaf surfaces; **D**. Corolla, front view; **E**. Flower; **F**. Longitudinally opened calyx; **G**. Longitudinally opened corolla; **H**. Ovary and Pistil; **I**. Stamens; **J**. Ovary; **K**. Open capsule showing seed. (**A–K**) photographed by L. Zhang. (**A**) *Hai-Feng Cao CAOHF140*. (**B–K**) *L. Zhang 20180607ZL*.

Fig. 5, Suppl. material 1: fig. S4

##### Type.

China • Sichuan: Aba Tibetan and Qiang Autonomous Prefecture, Songpan County, Huanglong Township, Danyunxia Scenic Area, 2566 m, 32.75223594, 103.96192293, 14 July 2025, *Hai-Feng Cao CAOHF140* (holotype: CSH 0222530!, isotype: CSH 0222531!, KUN!, PE!, SMCM!).

##### Diagnosis.

*G. tingnongiana* differs from *G. filisepala*, *G. baoxingensis*, *G. vandellioides* and *G. rubicunda* in that the corolla is yellowish-green striped externally, pale purple or pink internally (rarely yellowish white), with blackish-purple spots in the throat and plicae and the smaller seeds (0.7–1.0 mm vs 1.0–1.5 mm in diameter). It can be distinguished from *G. filisepala*, *G. baoxingensis* and *G. vandellioides* by usually having a main erect stem (vs. stems ascending, much branched from base), obovate, obovate-elliptic or oblong stem leaf blades (vs. ovate, broadly ovate to subcordate), and the earlier flowering and fruiting period (April to July vs. June to September). It can be distinguished from *G. rubicunda* by the smaller corolla size (0.9–1.5 cm vs. 1.5–6 cm long) and the shorter filaments (4–5 mm vs. 7–15 mm long).

##### Description.

***Annual herbs***, 5–22 cm tall. ***Taproot*** cylindrical, slender and delicate, surface yellowish-white. ***Stem erect*** with a main stem, rarely ascending, purplish-red, pale brown or green, smooth, with several slender ridges, simple or little branched from base or middle. ***Basal leaf blades*** 6–37 × 4–25 mm, obovate, obovate-oblong, spatulate or ovate, apex obtuse, margin smooth or sparsely ciliate, both surfaces glabrous, 3–5 distinct veins, petioles 2–10 mm long. ***Stem leaves*** nearly sessile or with a 1–2 mm long petiole, opposite leaves with petiole bases fused into a short sheath ca 0.5–1 mm long, leaf blades spreading, spaced apart, much shorter than internodes, becoming smaller and narrower toward the top of the stem; middle and lower stem leaf blades 8–15 × 4–11 mm, obovate or obovate-elliptic; upper stem leaf blades 6–10 × 1–4 mm, oblong, with bases slightly connate into a tube, margins ciliate, both surfaces glabrous, midvein prominent, lateral veins indistinct, apex obtuse or abruptly acute. ***Flowers*** numerous, solitary at branch tips. ***Pedicels*** 2–15 mm long, purplish red or pale brown, rarely yellowish green, slender, smooth, with fine longitudinal ridges, glabrous. ***Calyx*** 6–8 mm long, narrowly campanulate; calyx tube membranous, purplish-red or yellowish-green, 4–5 mm long; lobes filiform, 2.0–3.5 mm long, purplish red or green, apex acuminate, margin smooth, midvein distinct. ***Corolla*** 10–15 mm long, narrowly obconic to funnelform, inside pale purple, pink or subwhite, with pale yellow-green base, and with blackish-purple spots in throat and plicae, outside with broad yellowish-green stripes; lobes 2.5–3.5 mm long, ovate, apex obtuse-rounded, margin entire; plicae 1.5–2.5 mm long, regular, triangular, slightly shorter than lobes, apex acute, margin entire. ***Stamens*** inserted slightly below middle of corolla tube, equal; filaments 4–5 mm long, linear; anthers 1–1.2 mm long, narrowly ellipsoid. ***Ovary*** 3.5–4 mm long, narrowly ellipsoid, apex obtuse, base gradually narrowing; gynophore 2–3 mm long. ***Style*** ca 1.5–2.2 mm long, linear: stigma 2-lobed, lobes linear, apex revolute. ***Capsule*** 5–6.5 × 2.5–4 mm, oblong or elliptic, enclosed or exposed, apex obtuse-rounded, with broad wing, lateral margins with narrow wings; gynophore 3–6 mm long; ***Seeds*** 0.7–1.0 mm long, pale brown, glossy, ellipsoid, surface densely reticulate.

##### Phenology.

Flowering and fruiting from April to July.

##### Distribution and habitat.

This new species grows in Huanglong and Baiyang Township, Songpan County, Aba Tibetan and Qiang Autonomous Prefecture, Sichuan, China, at an elevation 1300–2566 m; and, in Pingwu and Beichuan Qiang Autonomous County, and Anzhou District, Mianyang City, Sichuan, China, at an elevation 1209–2500 m (Fig. 1).

##### Etymology.

The specific epithet honors the late Prof. Tingnong Ho, a renowned taxonomist who made significant contributions to the global classification of Gentianaceae.

##### Vernacular name.

Chinese mandarin: ting nong long dan (廷农龙胆).

##### Additional specimens examined.

China • Sichuan: Aba Tibetan and Qiang Autonomous Prefecture, Songpan County, Huanglong Township, Danyunxia Scenic Area, 2471 m a.s.l., 7 June 2018, *Lei Zhang 20180607ZL* (CSH 0210095!, PE!). • Aba Tibetan and Qiang Autonomous Prefecture, Songpan County, Huanglong Township, On the road from Shuanghe Village to 2199 m, 32.75223594°N, 103.96192293°E, 30 May 2023, *Peng-Cheng Fu Fu2023003* (LYUH!). • Aba Tibetan and Qiang Autonomous Prefecture, Songpan County, Baiyang Township, on the road from Maming Village to Wangye Temple, in the grass along the valley roadside, 1300 m, 18 May 1962, *Anonymous 0624* (CDBI CDBI0115731!). • Mianyang City, Pingwu County, At the foot of Xuebaoding Mountain, 21 May 2019, *Lei Zhang 2019ZL0505* (CSH 0210093!, PE!). • Mianyang City, Pingwu County, Daqiao Town, Daan Village, 2036 m, 32.33972222°N, 104.27027778°E, 22 April 2021, *Yuan Zou 2021ZY01* (CSH 0210092!, KUN 1653511!, PE!, SMCM!). • Mianyang City, Pingwu County, Baima Tibetan Township, Youqigou in the Baihegou, 2500 m, 7 July 1958, *H.L.Tsiang (Xinglin Jiang) 10748* (IBSC 0490213, image!; NAS NAS00077556, image!; SZ 00047819!; PE 00073557 & PE 00073558!). • Mianyang City, Beichuan Qiang Autonomous County, Kaiping Township, Near Niuxin Mountain within the Giant Panda Protection Area, between Baichikou and Renzhongling, 1209 m, 32.04645985°N, 104.35880501°E, 17 May 2022, *Hong Jiang 20220517-B01* (CSH 0210094!, KUN 1653510!, PE 02402079!, SMCM!). • Mianyang City, Beichuan Qiang Autonomous County, Piankou Township, Zhulingou, ca 1700 m, 28 May 2007, *Dahai Zhu 3832* (WCSBG 017528 & 017529!; PE 02103674!). • Mianyang City, Anzhou District, Qianfo Town, Mao’ergou, ca 1400 m, 23 May 2007, *Dahai Zhu 3710* (WCSBG 017526 & 017527, image!; PE 02104226 & 02104227!).

##### Preliminary conservation status.

*Gentiana tingnongiana* is distributed in Aba Tibetan and Qiang Autonomous Prefecture and Mian Yang City. Based on our field survey, the species has ten distribution sites. Its extent of occurrence (EOO) is 2,675.441 km^2^, and its area of occupancy (AOO) is 32 km^2^. The habitat conditions are good. Therefore, G. tingnongiana should be considered as Vulnerable (VU B1abiii; B2abiii) (IUCN 2024).

##### Notes.

The new species *Gentiana tingnongiana* is assigned to Ser. *Rubicundae* of Sect. *Chondrophyllae* by virtue of its filiform calyx lobes, and is morphologically similar to several species in this series. Nevertheless, it possesses a set of distinctive morphological characters that readily distinguish it from its closely related congeners: its corolla is usually pale purple, pink or subwhite, rarely pale blue-white, with numerous black-purple spots densely distributed in a ring-shaped pattern on the inner throat; spots are also conspicuously scattered on the inside of the plicae, and occasionally on the lower parts of the corolla lobes. In contrast, the inner corollas of other related species are consistently purple or pale blue, with immaculate plicae. *G. tingnongiana* typically has a distinct main stem, rarely lacking one. Among its allies, *G. rubicunda* also bears an evident main stem, while *G. filisepala*, *G. baoxingensis*, *G. vandellioides* and its variety *G. vandellioides* var. *biloba* are all devoid of a main stem. Besides the differences in corolla color and spots, *G. tingnongiana* is easily differentiated from *G. rubicunda* by its distinctly smaller flowers. The upper cauline leaves of *G. tingnongiana* are oblong, relatively narrow and elongated, with ciliate margins. In comparison, the cauline leaves of *G. baoxingensis*, *G. filisepala* and *G. vandellioides* are ovate or subcordate. The leaf margins of *G. baoxingensis* are conspicuously hirsute; those of *G. filisepala* are usually glabrous or occasionally sparsely ciliate and the margins of *G. vandellioides* and its variety *G. vandellioides* var. *biloba* are usually smooth and sometimes ciliolate.

#### Gentiana shangwui

Taxon classificationPlantaeGentianalesGentianaceae

H.F.Cao & L.Zhang sp. nov.

26BAE180-0338-5319-9EEF-77BA68B2BE3B

urn:lsid:ipni.org:names:77380713-1

**Figure 6.**
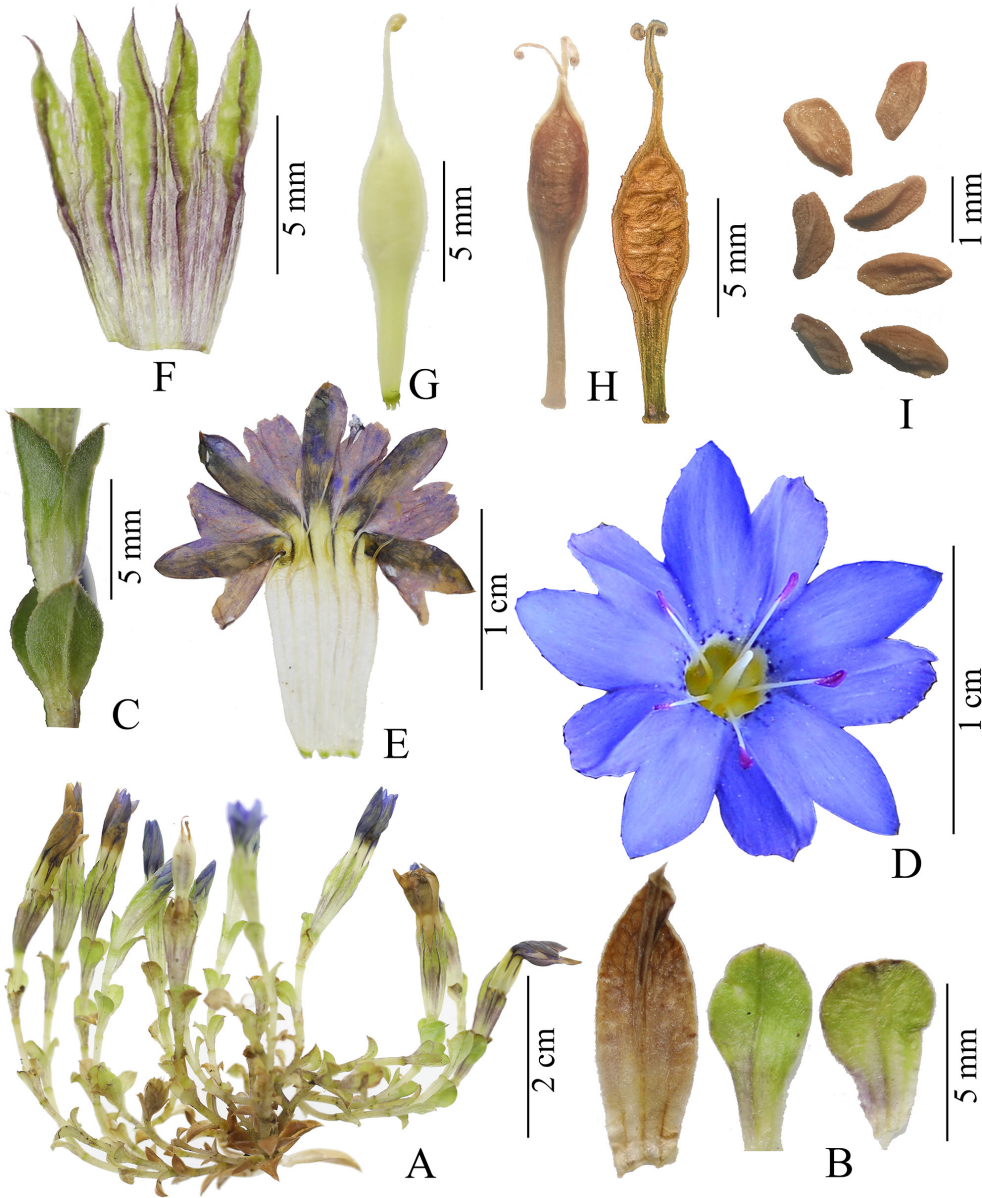
*
Gentiana shangwui* H.F.Cao & L.Zhang. **A**. Plant; **B**. Leaves; **C**. Stem leaves; **D**. Corolla, front view; **E**. Longitudinally opened corolla; **F**. Longitudinally opened calyx; **G**. Ovary & pistil; **H**. Fruits; **I**. Seeds. (**A–I**) photographed by H.F. Cao. (**A–C**) *X. D. Huang Emily 202237*. (**D–I**) *L. Zhang 2018ZL0727*.

Fig. 6, Suppl. material 1: fig. S5

##### Type.

China • Sichuan: Aba Tibetan and Qiang Autonomous Prefecture, Wenchuan County, Wolong Town, Balang Mountain, 4000 m, 27 July 2018, *Lei Zhang 2018ZL0727* (holotype: CSH!, isotype: PE 02402080!, KUN!).

##### Diagnosis.

*Gentiana shangwui* is morphologically similar to *G. nanobella* and *G. piasezkii*, but can be easily distinguished from them by having hirsute (sometimes glabrescent) leaves, and a 4–6 mm long style (vs. 1.2–2 mm long in *G. nanobella* and 2–4 mm long in *G. piasezkii*). It can be distinguished from *G. nanobella* by its slightly obovate-elliptic, 6–6.5 mm long capsules which are conspicuously narrowly winged along the sutures at the apex (vs cylindrical to narrowly ellipsoid, 10–12 mm long, not winged), a minutely and densely reticulate seed coat (vs. seed edges several, irregular, and spongy). It can be further distinguished from *G. piasezkii* by the suborbicular leaf blades with basal leaves 3–6.5 × 2–3 mm and stem leaves 2–4 × 2–3.5 mm (vs. leaf blade lanceolate, narrowly elliptic, rarely ovate, basal leaves involucriform, 17–20 × 4–6 mm and stem leaves 5–9 × 2–3 mm), the 2.5–3.3 mm long corolla lobes (vs. 4–5 mm long) and the stamens inserted in the apical part of corolla tube (vs. at middle of corolla tube).

##### Description.

***Annual herbs***, 2–6 cm tall. ***Stems*** yellowish-green or purplish-red and densely papillate, ascending to erect, branched from base. ***Basal leaves*** withered at anthesis, 3–6.5 × 2–3 mm, usually slightly larger than upper stem leaves, leaf blade oblong-ovate or ovate-lanceolate, apex rounded or acute, both surfaces glabrous, margin smooth to ciliolate and cartilaginous, sessile or with petiole up to 1.5 mm long. ***Stem leaves*** green or purplish-red, opposite, petiole 0.5–2.5 mm long, base connate into a 0.5–1 mm long tube, hirsute; leaf blade widely spaced, apex rounded and cuspidate, both surfaces puberulent and glabrescent, or sometimes abaxially puberulent on the midvein and adaxially glabrous, margin smooth to ciliolate and cartilaginous, midvein abaxially distinct; lower stem leaves 2–4 × 1.0–3.1 mm, ovate-lanceolate to suborbicular; middle and upper stem leaves 2–4 × 2–3.5 mm, suborbicular, larger toward upper stem, shorter to longer than internodes. ***Flowers*** few to many. ***Pedicels*** yellowish-green or slightly purplish-red and densely papillate, 1–6 mm long, usually surrounded by upper leaves, rarely exposed. ***Calyx*** tubular to narrowly obconic, 9–12 mm long, outside puberulent and glabrescent; tube tubular, 7–9 mm long; lobes triangular, 1.8–2.5 mm long, margin narrowly membranous and smooth, apex acute and cuspidate, midvein strongly keeled and decurrent into conspicuous wings of calyx tube. ***Corolla*** dark blue or blue-purple, with yellow-white base, throat with dark blue-purple spots or blotches, rarely immaculate; outside upper tinged blackish-blue; middle of corolla tube bright yellow or pale yellow, with fine blackish-blue obconic streaks, tubular to narrowly funnelform, 15–20 mm long; lobes ovate, 2.5–3 × 1.5–2 mm, margin entire, apex obtuse; plicae ovate, regular or slightly oblique, 1.5–2.5 × 2–2.8 mm, margin erose, apex obtuse. ***Stamens*** inserted at apical part of corolla tube, about 9–12 mm from the base and 4.5–5.5 mm from the upper part of the tube, equal; filaments filiform, 4.5–5.5 mm long, white; anthers purplish red, pink, or yellow, 0.8–1.2 mm long, ellipsoid. ***Ovary*** oblanceolate, 4–6 mm long, apex acute, base gradually narrowing. ***Style*** 4–6 mm long, linear, 2-lobed, lobes revolute. ***Capsule*** 6–6.5 mm long, narrowly obovoid to narrowly ellipsoid, apex long attenuate, with extremely narrow, translucent wings along both lateral margins at both ends, absent in the middle, base obtuse-rounded; gynophore up to 8 mm long. ***Seeds*** light brown, ellipsoid, 1.0–1.5 mm long, 0.4–0.8 mm wide, coat minutely and densely reticulate.

##### Phenology.

Flowering and fruiting from July to September.

##### Distribution and habitat.

*Gentiana shangwui* is currently known only from its type locality in Wolong Town, Wenchuan County, and Siguniangshan Town, Xiaojin County, Aba Tibetan and Qiang Autonomous Prefecture City, Sichuan, China. It grows in alpine meadows of Balang Mountain, at an elevation of 3800–4500 m (Fig. 1).

##### Etymology.

This species is dedicated to the late Prof. Shangwu Liu in recognition of his outstanding expertise in Gentianaceae and his generous guidance provided to the first author (H.F.Cao).

##### Vernacular name.

Chinese mandarin: shang wu long dan (尚武龙胆).

##### Preliminary conservation status.

*Gentiana shangwui* is currently known only from its type locality in Siguniangshan Town, Xiaojin County, Aba Tibetan and Qiang Autonomous Prefecture City, Sichuan, China. Only four specimens, collected from 2015 onwards, exist, but since these are from a nearby locality, it is impossible to calculate the Extent of Occurrence (EOO). The Area of Occupancy (AOO) is 4 km^2^, and the species is known only from a single location. These conditions qualify the species for the Critically Endangered category under IUCN Criterion B2 and subcriterion B2a.

*Gentiana shangwui* occurs on Balang Mountain within Wolong Nature Reserve, where the habitat is well preserved and suitable for the survival of the species. Therefore, this species should be assessed as Endangered (EN B2abiii) (IUCN 2024).

##### Additional specimens examined.

China • Sichuan: Aba Tibetan and Qiang Autonomous Prefecture, Xiaojin County, Siguniangshan Town, Balang Mountain, 3800 m, 5 August 2022, *Xiaodong Huang Emily202237* (CSH 0210079!, SMCM!). • Aba Tibetan and Qiang Autonomous Prefecture, Xiaojin County, Siguniangshan Town, Balang Mountain, 30.91590833°N, 102.88571944°E, 4500 m, 14 September 2015, *FLPH Sichuan Expedition 153018* (PE 02284225!; PE 02284304!). • Aba Tibetan and Qiang Autonomous Prefecture, Xiaojin County, Siguniangshan Town, Balang Mountain, 30.917912°N, 102.885317°E, 4414 m, 12 August 2018, *W.G.Sun, X.G.Ma et al. FSC-219* (KUN!).

##### Notes.

The most diagnostic characters of the new species *Gentiana shangwui* are its long style (4–6 mm long), cauline leaves pubescent on both surfaces, and the yellow or orange-yellow corolla tube at the middle part; these features readily distinguish it from other closely related congeners. *Gentiana shangwui* is often misidentified as *G. nanobella* due to their great similarities in overall morphology and foliar characters, but differs in having oblanceolate capsules with narrowly winged apices and seeds with fine reticulations (vs. linear-cylindric capsules without narrowly winged apices and vesicular seeds in *G. nanobella*). It also shares certain similarities with *G. piasezkii* in capsule morphology and the characters of corolla and calyx, yet is easily distinguished from the latter by its smaller plant size, deep blue corolla with irregularly short linear maculations, broadly ovate cauline leaves, and smaller basal leaves. *G. winchuanensis* is distinctly different from *G. shangwui* in having larger corollas and broadly ovate cauline leaves. Based on the protologue and photocopied images of the holotype of *G. aphrosperma*, this taxon is characterized by linear-cylindric capsules without apical wings and vesicular seeds, which are consistent with the diagnostic features of *G. nanobella*; thus, it is appropriate to reduce *G. aphrosperma* to a synonym of *G. nanobella*. Field observations revealed that *G. nanobella* exhibits considerable morphological variations in corolla color, corolla size, presence or absence of maculations, and seed size among different populations, whereas its capsule, leaf and corolla morphologies are relatively stable. Such morphological variations of *G. nanobella* merit further investigation.

## Supplementary Material

XML Treatment for Gentiana tingnongiana

XML Treatment for Gentiana shangwui

## Additional information

### Artificial Intelligence (AI) use

The authors accept full responsibility for the content of the manuscript, including the disclosure of any use of AI.

No AI tools were used in the preparation of this manuscript.

### Author ORCIDs

H.-F. Cao https://orcid.org/0000-0001-5489-8504

J. Cai https://orcid.org/0000-0003-1627-3700

J.-D. Ya https://orcid.org/0000-0003-3389-1412

P.-C. Fu https://orcid.org/0000-0002-6201-0808

L. Zhang https://orcid.org/0000-0001-5301-4658

### Data availability

All of the data that support the findings of this study are available in the main text or Supplementary Information.

## Supplementary materials

Supplementary material 1 Species and GenBank accession numbers https://doi.org/10.3897/phytokeys.275.190176.suppl1 Data type docx Explanation note Species and GenBank accession numbers for the ITS region used in this study. https://binary.pensoft.net/file/1659743This dataset is made available under the Open Database License (http://opendatacommons.org/licenses/odbl/1.0/). The Open Database License (ODbL) is a license agreement intended to allow users to freely share, modify, and use this Dataset while maintaining this same freedom for others, provided that the original source and author(s) are credited. Hai-Feng Cao, Liang-Hong Ni, Jie Cai, Ji-Dong Ya, Gan Li, Yue-Qing Luo, Peng-Cheng Fu, Lei Zhang
