# Supplementary material for: Gentiana tingnongiana and G. shangwui, two new species of Gentiana (Gentianaceae) from the eastern Qinghai-Tibet Plateau, Sichuan province, China
Source: PhytoKeys. 2026 May 27;275:97–113. doi: 10.3897/phytokeys.275.190176 (PMC13234560; doi:10.3897/phytokeys.275.190176)
Supplement: Supplementary material 1 — Species and GenBank accession numbers [file phytokeys-275-097_article-190176__-s001.docx]

Table S1 Material studied for morphological comparison between *G. tingnongiana*, *G. filisepala*, *G. baoxingensis*, *G. vandellioides* and *G. rubicunda*

| Species | Locality | Altitude | Latitude/Longitude | Collection Date | Collector | Collection Number | Herbarium Code or Specimen Number |
| --- | --- | --- | --- | --- | --- | --- | --- |
| ***G. filisepala*** | Baoxing, Ya 'an City, Sichuan, CHINA. | 3300 m |  | 21-Jun-1954 | Z.P.Song | 38665 | SZ 00047815! holotype & 00047814! isotype |
|  | Baoxing, Ya 'an City, Sichuan, CHINA. CHINA.CHINA.Qiaoqi Town |  |  | 9-May-1938 | Qu (G.L.Qu) | 6329 | PE 00073292 & 00073292! |
|  | Baoxing, Ya 'an City, Sichuan, CHINA. | 3000 m |  | 11-Aug-1936 | G.L.Qu | 3547 | PE 00073289-00073291!; WUK WUK0318908, image! paratype |
|  | Baoxing, Ya 'an City, Sichuan, CHINA. |  |  | Jun-Aug-1939 | X.Y.Hu | 1317 | SZ 00047816! paratype |
|  | Baoxing, Ya 'an City, Sichuan, CHINA. |  |  |  | Z.X.Qu | 3547 | SZ 00047818! |
|  | Baoxing, Ya 'an City, Sichuan, CHINA. | ca.2461 m |  | 14 June 2019 | X.W.Sun | 2019SXW01 | SMCM! |
|  | Baoxing, Ya 'an City, Sichuan, CHINA. | 3740 m |  | 28-Jul-1936 | T.H.Tu | 4519 | PE 00073288! |
|  | Baoxing, Ya 'an City, Sichuan, CHINA. |  |  | 1933 | Students | 4519 | CQNM! |
|  | Qiaoqi Town, Baoxing, Ya 'an City, Sichuan, CHINA. |  |  | 5-Jul-1978 | Baoxing Plant Survey Team | 78-0627 | SM! |
|  | Muping Town, Baoxing, Ya 'an City, Sichuan, CHINA. | 3318 m | 30.73985 N, 102.84225 E | 12-Jul-2022 | Q.Yu | YQSC003 | PE02428604! |
|  | Sanhe Township, Kangding County, Ganzi Tibetan Autonomous Prefecture, Sichuan, CHINA. | 2782 m |  | 6-Jun-2019 | Q.Yu, J.Xiong | CIBYQ069B008 | KUN 1503984! |
|  | Sanhe Township, Kangding County, Ganzi Tibetan Autonomous Prefecture, CHINA. | 2220-2702 m |  | 15-Aug-2017 | Q.Yu, X.H.Xiong, L.L. Li | THP-KD-4973 | CDBI 0249187 & 0249188! |
|  | Pengta Township, Kangding County, Ganzi Tibetan Autonomous Prefecture, CHINA. | 3932 m |  | 3-Jun-2017 | Q.Yu, C.C.Zheng | THP-KD-0291 | CDBI 0249189 & 0249190! |
| ***G. baoxingensis*** | Baoxing, Sichuan, CHINA. | 4000 m |  | 12-Aug-1936 | G.L. Qu (K.L. Chu) | 3553 | IBSC image! holotype; CDBI not seen, PE! isotype |
| ***G. vandellioides*** | Fang county (Fang), Shiyan City, Hubei (Hupeh), CHINA. |  |  |  | Henry | 6871 | K, lectotype; E00001792, NY00297717, P00350692, isolectotypes, all image! |
|  | Fang county (Fang), Shiyan City, Hubei (Hupeh), CHINA. |  |  |  |  | 6738 | BM!, GH!, K!, NY!, Syntypes, all image! |
|  | Shennongjia Forestry District, CHINA. | 2100 m | 31.71N, 110.46E | 16-Jul-2025 | P.C.Fu | Fu2025040 | LYUH! |
| ***G. vandellioides*** **var. *biloba*** | Chengkou Xian (Tchenkeoutin), Sichuan (Setchuen), CHINA. |  |  |  | R.P.Farges | 1106 | P00350698, lectotype; K, P00350699-00350701, UPS, isolectotypes, all image! |
|  | Chengkou Xian (Tchenkeoutin), Sichuan (Setchuen), CHINA. |  |  |  | R.P.Farges | 1243 | GH, P00350702, Syntypes, all image! |
|  | Chengkou Xian (Tchenkeoutin), Sichuan (Setchuen), CHINA. |  |  | 7-Jul-1958 | T.L.Dai | 101185 | CDBI!, HNWP!, SZ! |
|  | Anle Village, Beiping Township, Chengkou Xian (Tchenkeoutin), Sichuan (Setchuen), CHINA. | 2300 m |  | 22-May-2024 | S.R.Yi | YSR3051 | CGMC! |
|  | Dazhaiyan Village, Lantian Township, Chengkou Xian (Tchenkeoutin), Sichuan (Setchuen), CHINA. | 1780 m |  | 15-Jul-2024 | S.R.Yi | YSR3430 | CGMC! |
| ***G. rubicunda*** | Tcheng-fong-chan (Chengfengshan), Yanjin county, Yunnan, CHINA. |  |  | May 1882 | Delavay | Gent.n. 8 | P, lectotype; E, K, P, UPS, isolectotypes, all image! |
|  | Io-chan, Qiaojia, Yunnan, CHINA. | 3200 m |  | Oct-1912 | E.E.Maire | 429 | E 00001767–00001769, BM, all image! |
|  | Ya 'an City, Sichuan, CHINA. | 600–1160 m |  | 1888 | G.Li | 1888 | HNWP! |
|  | Ya 'an City, CHINA. |  |  | 1987 | G.Li | 122-1B | HNWP! |
|  | Xiaozhaizigou, Beichuan County, Mianyang City, Sichuan, CHINA. |  |  | 13-Jul-2017 | L.Zhang | 2017ZL0713 | SMCM! |
|  | Pingwu county, Mianyang City, Sichuan, CHINA. |  |  | 3-Jun-1961 |  | 13137 | SM 716500589! |
|  | Omei Mountain, Emeishan county, Leshan City, Sichuan, CHINA. |  |  | 16-Feb-1942 | W.P.Fang | 18118 | PE00094672! |
|  | Omei Mountain, Emeishan county, Leshan City, Sichuan, CHINA. |  |  | 14-Sep-2014 | X.J.Li | CSH05810 | CSH 0040033! |
|  | Xiling Snow Mountain, Dayi county, Chengdu City, Sichuan, CHINA. | 3119 m |  | 4-Aug-2018 | H.F.Cao & L.Zhang | 2018CHF04 | CSH!, SMCM! |
|  | Dengshenggou, Wolong Town, Wenchuan County, Ganzi Tibetan and Qiang Autonomous Prefecture, Sichuan, CHINA. |  |  | 4-Aug-2022 | X.D.Huang | Emily202236 | SMCM! |
|  | Chengkou county, Chongqing (Sichuan), CHINA. |  |  | 30-May-1958 | T.L.Dai | 100741 | CDBI!, HNWP!, PE!, SZ! |
|  | Longshan County, Hunan: Xiangxi Tujia and Miao Autonomous Prefecture, Sichuan, CHINA. |  |  | 19-Apr-2012 | Y.Xiao, J.J.Zhou | LS-047 | CSH 0030522! |

Table S2 Material studied for morphological comparison between *G. shangwui, G. piasezkii*, *G. winchuanensis* and *G. nanobella*

| Species | Locality | Altitude | Latitude/Longitude | Collection Date | Collector | Collection Number | Herbarium Code or Specimen Number |
| --- | --- | --- | --- | --- | --- | --- | --- |
| ***G. winchuanensis*** | Weizhou District, Winchuan County, Sichuan: Aba Tibetan and Qiang Autonomous Prefecture, CHINA. | 2400 m |  | 30-Jul-1975 | Sichuan Veget. Exped. | 8522 | HNWP!, holotype; CDBI CDBI0116216 & 0116217!, PE 00088757!, isotypes |
|  | Maoxian County, CHINA. | 3280 m | 31.50N, 103.76E | 21-Jul-2025 | P.C.Fu | Fu2025056 | LYUH! |
| ***G. piasezkii*** | Shaanxi (Schensi), CHINA. |  |  | 1875 | Piasezky |  | LE holotype, not seen; PE00029366!, UPS image! , isotype; HNWP! photo.from LE |
|  | Wenjiagou Village, Majie Town, Wudu District, Longnan City, Gansu, CHINA. | 2356 m | 33°33.134′N, 104°53.5767′E | 9-Jun-2025 | H.F.Cao | CAOHF133 | CSH!, KUN!, SMCM! |
|  | Baiyangwan Village, Maying Town, Wudu District, Longnan City, Gansu, CHINA. | 2429 m | 33°37.8041′N, 104°53.0415′E | 9-Jun-2025 | H.F.Cao | CAOHF134 | CSH!, KUN!, SMCM! |
|  | jiuzhaigou scenic spot, Jiuzhaigou County, Aba Tibetan and Qiang Autonomous Prefecture, Sichuan, CHINA. | 3579 m | 32°43.5117′N, 103°49.9326′E | 12-Jul-2025 | H.F.Cao | CAOHF138 | CSH!, KUN!, SMCM! |
| ***G. nanobella*** | Lancangjiang-Nujiang (Mekong-Salween) divide, Yunnan, CHINA. | 3900 m |  | Aug-1914 | Forrest | 13220 | E, holotype; K, UPS, isotypes, all image! |
| ***G. nanobella*** | Baima Snow Mountain, Deqin County, Diqing Tibetan Autonomous Prefecture, Sichuan, CHINA. | 4459 m |  | 25-Aug-2018 | W.G.Sun, X.G.Ma et all | FSC-692 | KUN! |
|  | Geza Township, Shangri-La County, Diqing Tibetan Autonomous Prefecture, Sichuan, CHINA. |  | 31°54'54''N,  93°6'43''E | 3-Oct-2025 | H.F.Cao | CAOHF216 | KUN! |
|  | Balang Mountain, Wolong Town,, Wenchuan County, Aba Tibetan and Qiang Autonomous Prefecture City, Sichuan, CHINA. |  |  | 12-Aug-2020 | D.C.Meng | 2020MDC01 | SMCM! |
|  | Balang Mountain, Wolong Town, Wenchuan County, Aba Tibetan and Qiang Autonomous Prefecture City, Sichuan, CHINA. | 4414 m |  | 12-Aug-2018 | W.G.Sun, X.G.Ma et all | FSC-219 | KUN! |
|  | Kangding City, Sichuan,CHINA. | 4464 m |  | 8-Aug-2018 | W.G.Sun, X.G.Ma et all | FSC-79 | KUN! |
|  | Xiaojin County, Aba Tibetan and Qiang Autonomous Prefecture City, Sichuan, CHINA. | 4258 m |  | 16-Aug-2018 | W.G.Sun, X.G.Ma et all | FSC-313 | KUN! |
| ***G. nanobella***（**synonym**：***G. aphrosperma***） | Hsueh-po-ting (Hsioeh-shan), Songpan, N Sichuan (Sze-ch'uan), CHINA. | 4300 m |  | 1l August 1922 | H.Smith | 3420 | UPS, holotype, not seen; E, image! photo. from the holotype of UPS |

Table S3 GenBank accession numbers for the ITS region for *Gentiana* species and outgroups used in this study. *** newly sequenced.

| Species | GenBank Accession Number |
| --- | --- |
| *Crawfurdia angustata* C.B.Clarke | GU251013 |
| *G. anisostemon* C.Marquand | KT907609 |
| *G. arethusae* Burk. | KT907611 |
| *G. asclepiadea* L. | GU251025 |
| *G. asterocalyx* Diels | KT907613 |
| *G. austromontana* J.S.Pringle & Sharp | KT907615 |
| *G. bavarica* L. | KT907617 |
| *G. bredboensis* L.G.Adams | KT907622 |
| *G. calycosa* Griseb. | KT907625 |
| *G. cephalantha* Franch. | KT907627 |
| *G. crassula* Harry Sm. | KT907633 |
| *G. cruciata* L. | DQ398635 |
| *G. davidii* Franch. | KT907637 |
| *G. decumbens* Linnaeus f. | DQ398655 |
| *G. depressa* D. Don | GU251026 |
| *G. dolichocalyx* T.N.Ho | KT907640 |
| *G. duclouxii* Franch. | KT907642 |
| **G. filisepala* T.N.Ho | PX677828 |
| **G. filisepala* T.N.Ho | PX677829 |
| *G. futtereri* Diels & Gilg | DQ398658 |
| *G. gelida* M.Bieb. | KT907648 |
| *G. glauca* Pall. | KT907649 |
| *G. handeliana* Harry Sm. | KT907652 |
| *G. heleonastes* Harry Sm. | KT907653 |
| *G. hexaphylla* Maxim. ex Kusn. | KT907654 |
| *G. kaufmanniana* Regel & Schmalh. | DQ398649 |
| *G. lacerulata* Harry Sm. | KT907661 |
| *G. lhassica* Burk. | DQ398629 |
| *G. linearis* Froel. | KT907664 |
| *G. lineolata* Franch. | KT907665 |
| *G. lutea* L. | KT907668 |
| *G. macrophylla* Pall. | DQ398652 |
| *G. nanobella* C.Marquand | KF563956 |
| *G. newberryi* A.Gray | KT907678 |
| *G. nivalis* L. | KT907679 |
| *G. officinalis* Harry Sm. | DQ398639 |
| *G. olgae* Regel & Schmalh. | KT907681 |
| *G. olivieri* Griseb. | DQ398645 |
| *G. orbicularis* Schur | KT907682 |
| *G. oreodoxa* Harry Sm. | DQ398657 |
| *G. pedicellata* (Wall. ex D. Don) Griseb. | KT907689 |
| *G. piasezkii* Maxim. | KU512343 |
| *G. piasezkii* Maxim. | KT907691 |
| *G. praeclara* C. Marquand | KT907693 |
| *G. punctata* L. | KT907701 |
| *G. purpurea* L. | KT907702 |
| *G. robusta* King ex Hook. f. | DQ398643 |
| *G. rostanii* Reut. ex Verlot | KT907704 |
| *G. rubicunda* Franch. | KT907705 |
| **G. rubicunda* Franch. | PX677830 |
| **G. rubicunda* Franch. | PX677831 |
| *G. saponaria* L. | KT907708 |
| *G. scabra* Bunge | KT907709 |
| *G. schleicheri* (Vacc.) Kunz | KT907711 |
| **G. shangwui* H.F.Cao & L.Zhang | PX677825 |
| **G. shangwui* H.F.Cao & L.Zhang | PX677826 |
| **G. shangwui* H.F.Cao & L.Zhang | PX677827 |
| *G. sino-ornata* Balf.f. | KT907715 |
| *G. siphonantha* Maxim. ex Kusn. | KT907716 |
| *G. spathacea* Kunth | KT907717 |
| *G. spathulifolia* Maxim. ex Kusn. | KT907718 |
| *G. striolata* T.N.Ho | KT907723 |
| *G. szechenyii* Kanitz | KT907726 |
| *G. terglouensis* Hacq. | KT907731 |
| *G. tianschanica* Rupr. | KT907733 |
| *G. tibetica* King ex Hook. f. | DQ398640 |
| **G. tingnongiana* H.F.Cao & L.Zhang | PX677822 |
| **G. tingnongiana* H.F.Cao & L.Zhang | PX677823 |
| **G. tingnongiana* H.F.Cao & L.Zhang | PX677824 |
| *G. trichotoma* Kusn. | KT907734 |
| **G. vandellioides* Hemsl. | PZ298042 |
| *G. verna* L. | KT907684 |
| *G. verna* subsp. oschtenica (Kusn.) Halda | KT907684 |
| *G. waltonii* Burki. | DQ398626 |
| *G. walujewii* Regel & Schmalh. | DQ398646 |
| **G. winchuanensis* T.N.Ho | PZ298043 |
| *G. yunnanensis* Franch. | KT907740 |
| *Metagentiana australis* (Craib) T.N.Ho & S.W.Liu | GU251029 |
| *Tripterospermum alutaceifolium* (T.S.Liu & C.C.Kuo) J.Murata | GU251037 |

Table S4 The measured data of *Gentiana tingnongiana, G. filisepala, G. baoxingensis, G. vandellioides* and *G. rubicunda*

| species name | Specimen number | Leaf length (cm) | Leaf width (cm) | Leaf area (cm^2^) | Leaf margin hair length (mm) | Corolla radius (cm) | Corolla length (cm) |
| --- | --- | --- | --- | --- | --- | --- | --- |
| *G. tingnongiana* | 2021ZY01 | 0.905 | 0.286 | 0.171 | 0.122 | 0.671 | 1.281 |
| *G. tingnongiana* | 2021ZY01 | 0.844 | 0.312 | 0.158 | 0.121 | 0.684 | 1.125 |
| *G. tingnongiana* | 2021ZY01 | 0.887 | 0.321 | 0.189 | 0.190 | 0.628 | 1.171 |
| *G. tingnongiana* | 2019ZL0505 | 0.898 | 0.312 | 0.187 | 0.221 | 0.658 | 1.111 |
| *G. tingnongiana* | 2019ZL0505 | 1.003 | 0.312 | 0.234 | 0.191 | 0.731 | 1.218 |
| *G. tingnongiana* | 2019ZL0505 | 0.867 | 0.262 | 0.151 | 0.195 | 0.649 | 1.294 |
| *G. tingnongiana* | CAOHF140 | 0.966 | 0.314 | 0.195 | 0.19 | 0.729 | 1.097 |
| *G. tingnongiana* | CAOHF140 | 0.868 | 0.26 | 0.152 | 0.189 | 0.691 | 1.068 |
| *G. baoxingensis* | IBSC0489357 | 0.31 | 0.197 | 0.051 | 0.36 | 0.443 | 0.986 |
| *G. baoxingensis* | IBSC0489357 | 0.289 | 0.233 | 0.054 | 0.283 | 0.467 | 0.99 |
| *G. baoxingensis* | IBSC0489357 | 0.317 | 0.298 | 0.074 | 0.27 | 0.484 | 0.92 |
| *G. baoxingensis* | IBSC0489357 | 0.29 | 0.213 | 0.045 | 0.341 | 0.423 | 1.054 |
| *G. baoxingensis* | IBSC0489357 | 0.34 | 0.204 | 0.065 | 0.276 | 0.429 | 0.981 |
| *G. baoxingensis* | IBSC0489357 | 0.269 | 0.194 | 0.055 | 0.32 | 0.447 | 0.967 |
| *G. filisepala* | 00047815 | 0.698 | 0.343 | 0.165 | 0.239 | 0.795 | 1.76 |
| *G. filisepala* | 00047815 | 0.759 | 0.35 | 0.257 | 0.222 | 0.78 | 1.683 |
| *G. filisepala* | 00047815 | 0.798 | 0.324 | 0.236 | 0.238 | 0.859 | 1.687 |
| *G. filisepala* | 00073291 | 0.655 | 0.343 | 0.231 | 0.221 | 0.825 | 1.629 |
| *G. filisepala* | 00073291 | 0.676 | 0.333 | 0.185 | 0.191 | 0.851 | 1.874 |
| *G. filisepala* | 00073291 | 0.702 | 0.348 | 0.187 | 0.195 | 0.797 | 1.634 |
| *G. filisepala* | 00047818 | 0.759 | 0.324 | 0.238 | 0.215 | 0.856 | 1.827 |
| *G. filisepala* | 00047818 | 0.698 | 0.36 | 0.164 | 0.224 | 0.751 | 1.809 |
| *G. filisepala* | 00047818 | 0.715 | 0.344 | 0.213 | 0.237 | 0.802 | 1.654 |
| *G. vandellioides* | P00350698 | 0.819 | 0.583 | 0.331 | 0 | 0.624 | 1.305 |
| *G. vandellioides* | P00350698 | 0.842 | 0.435 | 0.342 | 0 | 0.709 | 1.411 |
| *G. vandellioides* | P00350698 | 0.957 | 0.48 | 0.25 | 0 | 0.671 | 1.324 |
| *G. vandellioides* | P00350701 | 0.975 | 0.414 | 0.395 | 0 | 0.651 | 1.453 |
| *G. vandellioides* | P00350701 | 0.98 | 0.5 | 0.323 | 0 | 0.642 | 1.218 |
| *G. vandellioides* | P00350701 | 0.893 | 0.534 | 0.385 | 0 | 0.617 | 1.284 |
| *G. vandellioides* | P00350699 | 0.812 | 0.452 | 0.331 | 0 | 0.687 | 1.42 |
| *G. vandellioides* | P00350699 | 0.795 | 0.401 | 0.352 | 0 | 0.683 | 1.231 |
| *G. rubicunda* | P00487014 | 0.876 | 0.506 | 0.28 | 0 | 0.872 | 2.244 |
| *G. rubicunda* | P00487014 | 0.81 | 0.378 | 0.257 | 0 | 0.883 | 2.177 |
| *G. rubicunda* | P00487014 | 0.914 | 0.582 | 0.338 | 0 | 0.85 | 2.277 |
| *G. rubicunda* | E00001764 | 0.882 | 0.465 | 0.265 | 0 | 0.904 | 2.479 |
| *G. rubicunda* | E00001764 | 0.833 | 0.489 | 0.252 | 0 | 0.862 | 2.323 |
| *G. rubicunda* | E00001764 | 0.919 | 0.477 | 0.362 | 0 | 0.892 | 2.731 |
| *G. rubicunda* | P00487613 | 0.803 | 0.559 | 0.345 | 0 | 0.916 | 2.191 |
| *G. rubicunda* | P00487613 | 0.93 | 0.539 | 0.334 | 0 | 0.892 | 1.941 |
| *G. rubicunda* | P00487613 | 0.883 | 0.565 | 0.347 | 0 | 0.893 | 2.352 |

Table S5 Analysis of variance of 6 morphological traits of *Gentiana tingnongiana, G. filisepala, G. baoxingensis, G. vandellioides* and *G. rubicunda*

| species name | Leaf length (cm) | Leaf width (cm) | Leaf area(cm^2^) | leaf margin hair length （mm） | Corolla radius (cm) | Corolla length (cm) |
| --- | --- | --- | --- | --- | --- | --- |
| *G. baoxingensis* | 0.30±0.03c | 0.22±0.04c | 0.06±0.01c | 0.31±0.04a | 0.45±0.02d | 0.98±0.04e |
| *G. filisepala* | 0.72±0.05b | 0.34±0.01b | 0.21±0.03b | 0.22±0.02b | 0.81±0.04b | 1.73±0.09b |
| *G. rubicunda* | 0.87±0.05a | 0.51±0.06a | 0.31±0.04a | 0.00±0.00d | 0.88±0.02a | 2.30±0.22a |
| *G. tingnongiana* | 0.90±0.05a | 0.30±0.02b | 0.18±0.03b | 0.18±0.04c | 0.68±0.04c | 1.17±0.09d |
| *G. vandellioides* | 0.88±0.08a | 0.47±0.06a | 0.34±0.04a | 0.00±0.00d | 0.66±0.03c | 1.33±0.09c |

Table S6 The measured data of *Gentiana shangwui*, *G. nanobella*, *G. piasezkii* and *G. winchuanensis*

| species name | Specimen number | Leaf length (cm) | Leaf width (cm) | Leaf area (cm^2^) | Corolla radius (cm) | Corolla length (cm) | Fruit wings (mm) |
| --- | --- | --- | --- | --- | --- | --- | --- |
| *G. piasezkii* | PE00029366 | 0.66 | 0.281 | 0.155 | 0.972 | 2.064 | 0.60 |
| *G. piasezkii* | PE00029366 | 0.741 | 0.303 | 0.154 | 0.876 | 2.208 | 0.51 |
| *G. piasezkii* | PE00029366 | 0.616 | 0.287 | 0.137 | 0.976 | 1.813 | 0.45 |
| *G. piasezkii* | K000843936 | 0.788 | 0.264 | 0.149 | 0.874 | 1.914 | 0.46 |
| *G. piasezkii* | K000843936 | 0.757 | 0.261 | 0.133 | 0.912 | 2.158 | 0.54 |
| *G. winchuanensis* | CDB10116216 | 0.511 | 0.257 | 0.105 | 0.981 | 2.583 | 0.42 |
| *G. winchuanensis* | CDB10116216 | 0.558 | 0.245 | 0.102 | 1.002 | 2.635 | 0.58 |
| *G. winchuanensis* | CDB10116216 | 0.463 | 0.364 | 0.126 | 0.995 | 2.556 | 0.47 |
| *G. winchuanensis* | CDB10116217 | 0.412 | 0.236 | 0.088 | 0.97 | 2.787 | 0.58 |
| *G. winchuanensis* | CDB10116217 | 0.492 | 0.381 | 0.132 | 0.941 | 2.863 | 0.48 |
| *G. winchuanensis* | CDB10116217 | 0.495 | 0.263 | 0.139 | 0.97 | 2.667 | 0.52 |
| *G. winchuanensis* | S.V.E.8522 | 0.565 | 0.329 | 0.125 | 0.997 | 2.758 | 0.49 |
| *G. winchuanensis* | S.V.E.8522 | 0.525 | 0.351 | 0.142 | 0.88 | 2.604 | 0.47 |
| *G. winchuanensis* | S.V.E.8522 | 0.498 | 0.387 | 0.121 | 0.974 | 2.627 | 0.51 |
| *G. nanobella* | E00001780 | 0.644 | 0.396 | 0.379 | 0.864 | 2.089 | 0 |
| *G. nanobella* | E00001780 | 0.713 | 0.315 | 0.257 | 0.911 | 2.198 | 0 |
| *G. nanobella* | E00001780 | 0.673 | 0.365 | 0.175 | 0.93 | 2.067 | 0 |
| *G. nanobella* | K000843543 | 0.749 | 0.383 | 0.212 | 0.842 | 2.118 | 0 |
| *G. nanobella* | K000843543 | 0.602 | 0.317 | 0.136 | 0.825 | 2.104 | 0 |
| *G. nanobella* | K000843543 | 0.561 | 0.401 | 0.168 | 0.796 | 2.022 | 0 |
| *G. nanobella* | K000843546 | 0.791 | 0.405 | 0.231 | 0.893 | 2.139 | 0 |
| *G. nanobella* | K000843546 | 0.649 | 0.388 | 0.183 | 0.877 | 2.07 | 0 |
| *G. nanobella* | K000843546 | 0.893 | 0.55 | 0.297 | 0.887 | 2.204 | 0 |
| *G. shangwui* | 2018ZL0727 | 0.397 | 0.238 | 0.063 | 0.772 | 1.592 | 0.45 |
| *G. shangwui* | 2018ZL0727 | 0.281 | 0.212 | 0.047 | 0.822 | 1.766 | 0.57 |
| *G. shangwui* | 2018ZL0727 | 0.3 | 0.236 | 0.057 | 0.853 | 1.597 | 0.44 |
| *G. shangwui* | Emily202237 | 0.324 | 0.284 | 0.068 | 0.833 | 1.673 | 0.48 |
| *G. shangwui* | Emily202237 | 0.297 | 0.258 | 0.09 | 0.875 | 1.769 | 0.52 |
| *G. shangwui* | Emily202237 | 0.368 | 0.267 | 0.091 | 0.789 | 1.836 | 0.42 |

Table S7 Analysis of variance of 6 morphological traits of *Gentiana shangwui*, *G. nanobella*, *G. piasezkii* and *G. winchuanensis*

| species name | Leaf length (cm) | Leaf width (cm) | Leaf area (cm^2^) | Corolla radius (cm) | Corolla length (cm) | Fruit wings (mm) |
| --- | --- | --- | --- | --- | --- | --- |
| *G. piasezkii* | 0.71±0.07a | 0.28±0.02bc | 0.15±0.01b | 0.92±0.05a | 2.03±0.17b | 0.51±0.06a |
| *G. winchuanensis* | 0.50±0.05b | 0.31±0.07b | 0.12±0.02b | 0.97±0.02a | 2.68±0.13a | 0.50±0.05a |
| *G. nanobella* | 0.70±0.10a | 0.40±0.04a | 0.23±0.09a | 0.87±0.04b | 2.11±0.05b | 0.00±0.00b |
| *G. shangwui* | 0.33±0.05c | 0.25±0.03c | 0.07±0.02c | 0.82±0.04b | 1.71±0.09c | 0.48±0.05a |


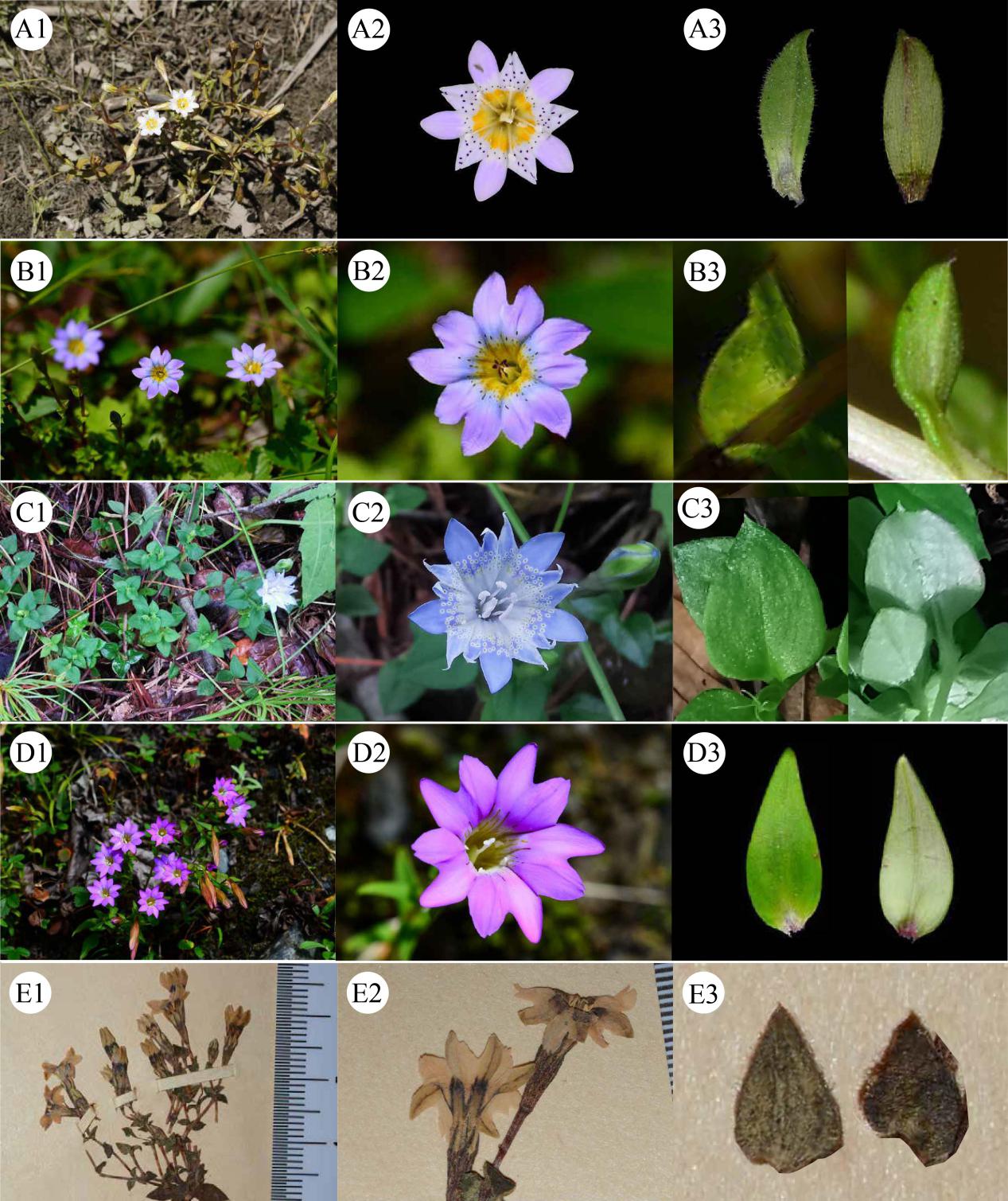


**Figure S1**. Morphological comparison of *Gentiana tingnongiana* and its closely related species. 1. Habit; 2. Flower, front view; 3. Leaves. **A** *G. tingnongiana*; **B** *G. filisepala*; **C** *G. vandellioides* var. *Biloba*; **D** *G. rubicunda*; **E** *G. baoxingensis*. **A1** *Hai-Feng Cao CAOHF140*, photographed by H.F. Cao; **A2-A3** *L. Zhang 20180607ZL*, photographed by L. Zhang; **B1-B3** *X.W.Sun 2019SXW01*, photographed by X.W.Sun; **C1-C3** *S.R.Yi YSR3051*, photographed by S. R. Yi; **D1-D3** *L. Zhang 2017ZL0713*, photographed by L.Zhang; **E1-E3** *G.L.Qu 3553* (IBSC), photographed by Y.P.Zeng


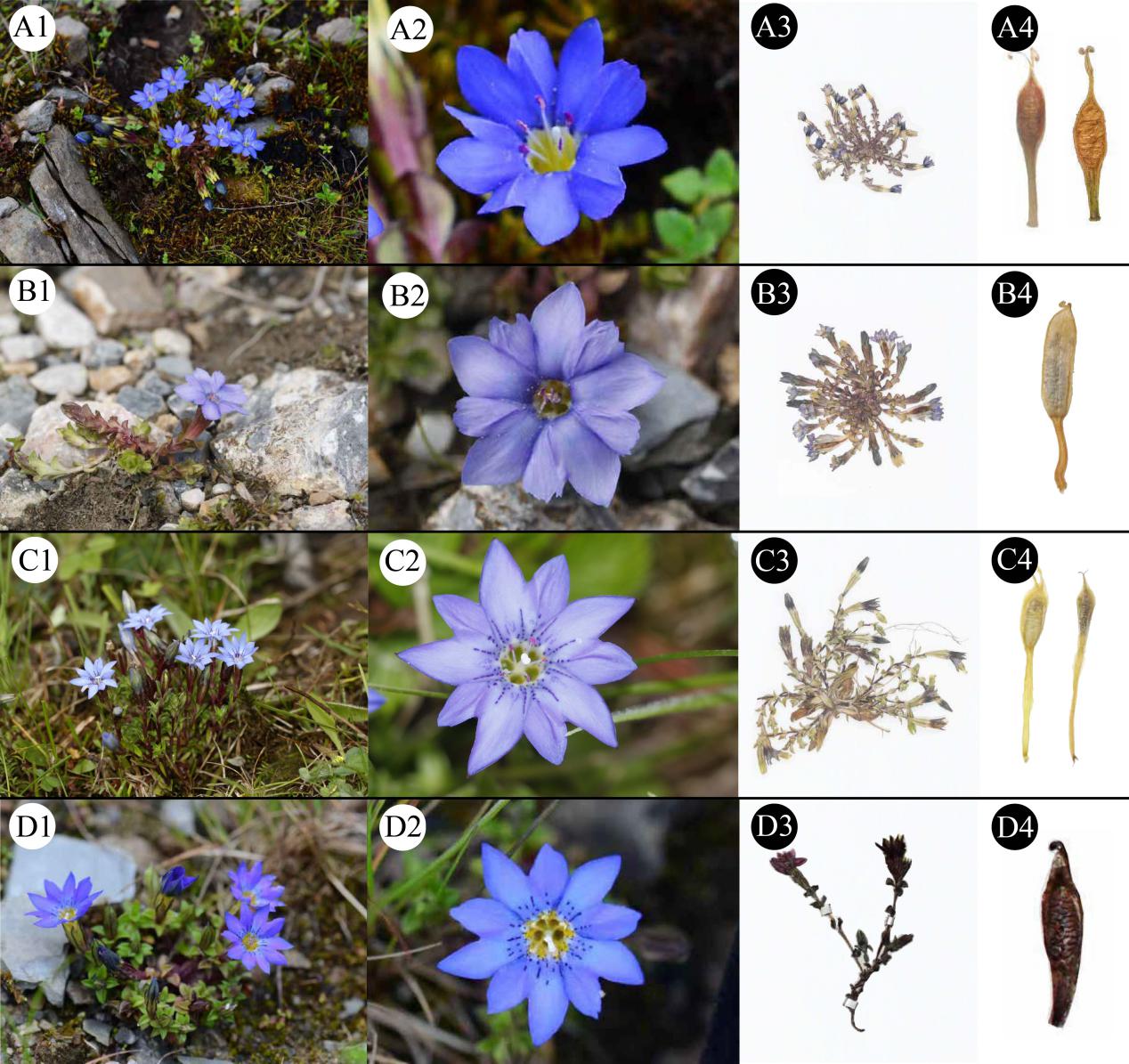


**Figure S2**. Morphological comparison of *Gentiana shangwui* and its closely related species. 1. Habit in nature; 2. Flower, frontal view; 3. Habit in dried condition; 4 Capsule. **A** *G. shangwui*; **B** *G. nanobella*; **C** *G. piasezkii*; **D** *G. winchuanensis.* **A1-A4** *L.Zhang 2018ZL0727*; **B1-B2** *H.F.Cao CAOHF216*; **B3-B4** *D.C.Meng 2020MDC01*; **C1-C4** *H.F.Cao CAOHF133*; **D1-D2** *P.C.Fu Fu2025056*; **D3-D4** *Sichuan Veget. Exped. 8522*; **A1-A2** photographed by L.Zhang; **A3-A4, B1-B4, C3-C4, D3-D4** photographed by H.F.Cao; **D1-D2** photographed by P.C.Fu


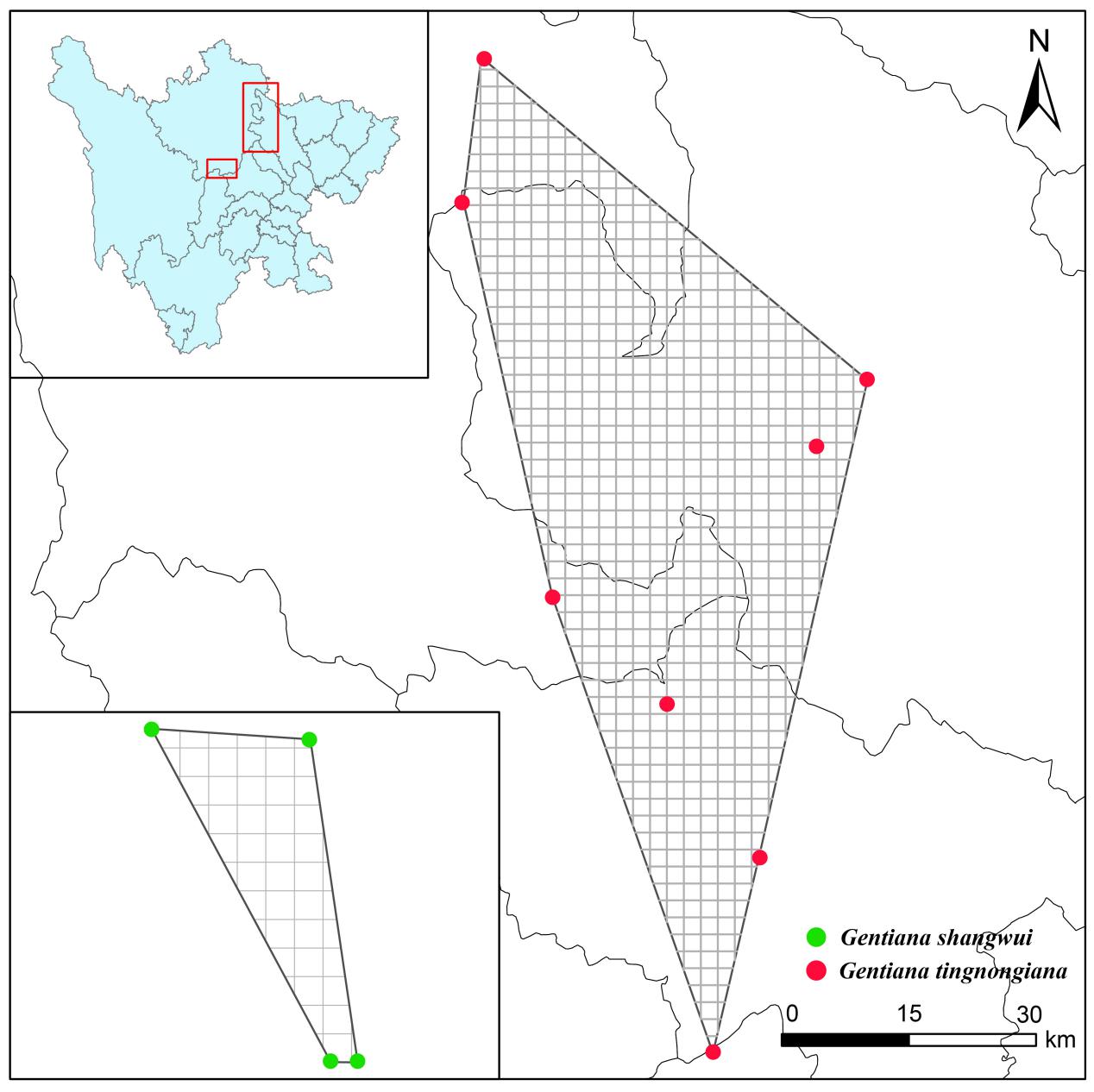


**Figure S3**. Distribution map of *Gentiana tingnongiana* (area: EOO=2675.441 km²; AOO=32 km²) and *G. shangwui* (area: AOO=4.000 km²).


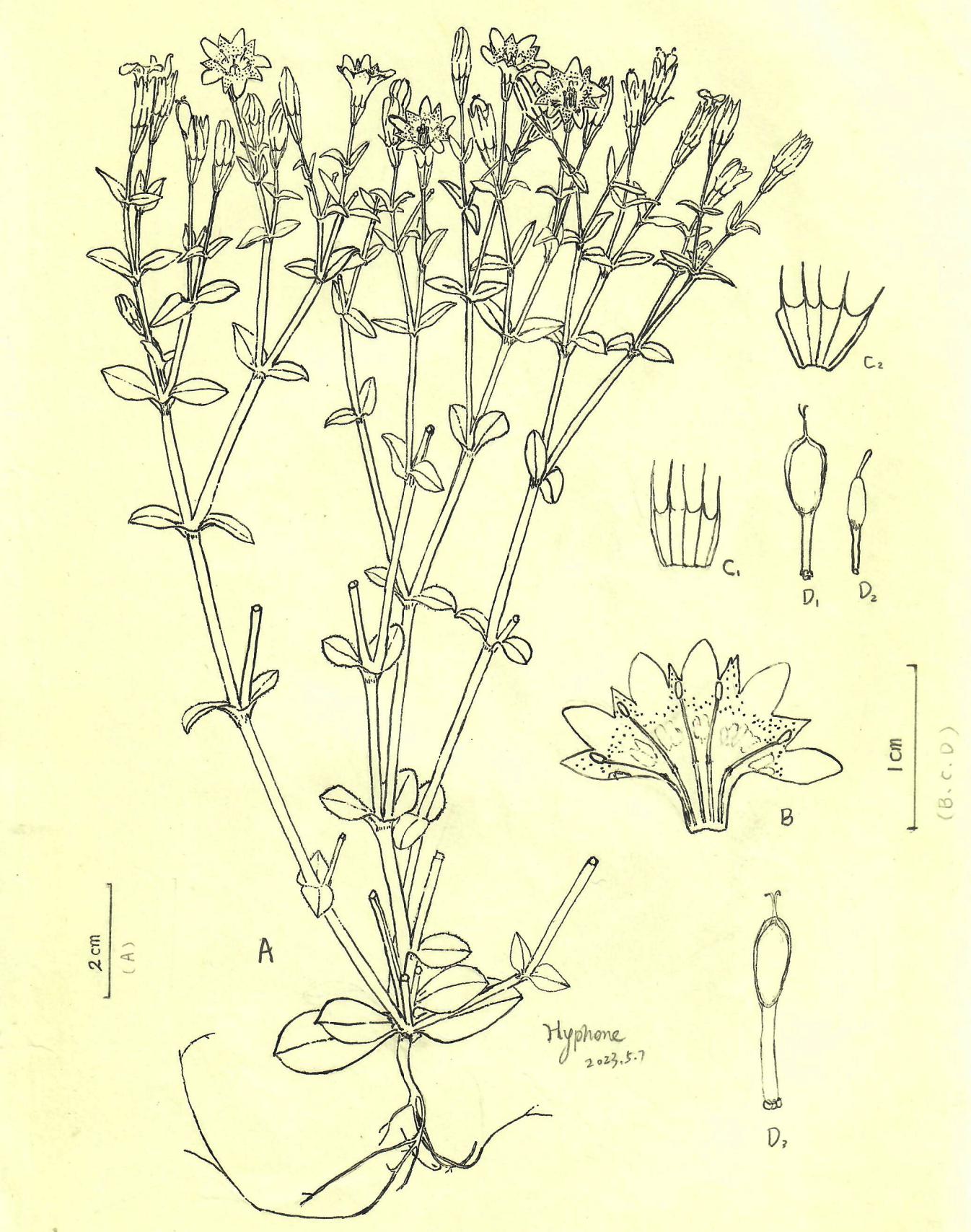


**Figure S4**. *Gentiana tingnongiana* H.F.Cao & L.Zhang **A** Habit; **B** Longitudinally opened corolla; **C1-C2** Longitudinally opened calyx; **D1-D3** Ovary and Pistil. Scale bars: 2 cm (**A**); 1 cm (**B**, **C1-C2**, **D1-D3**). Drawn by H.F.Cao based on *L.Zhang 20180607ZL*.


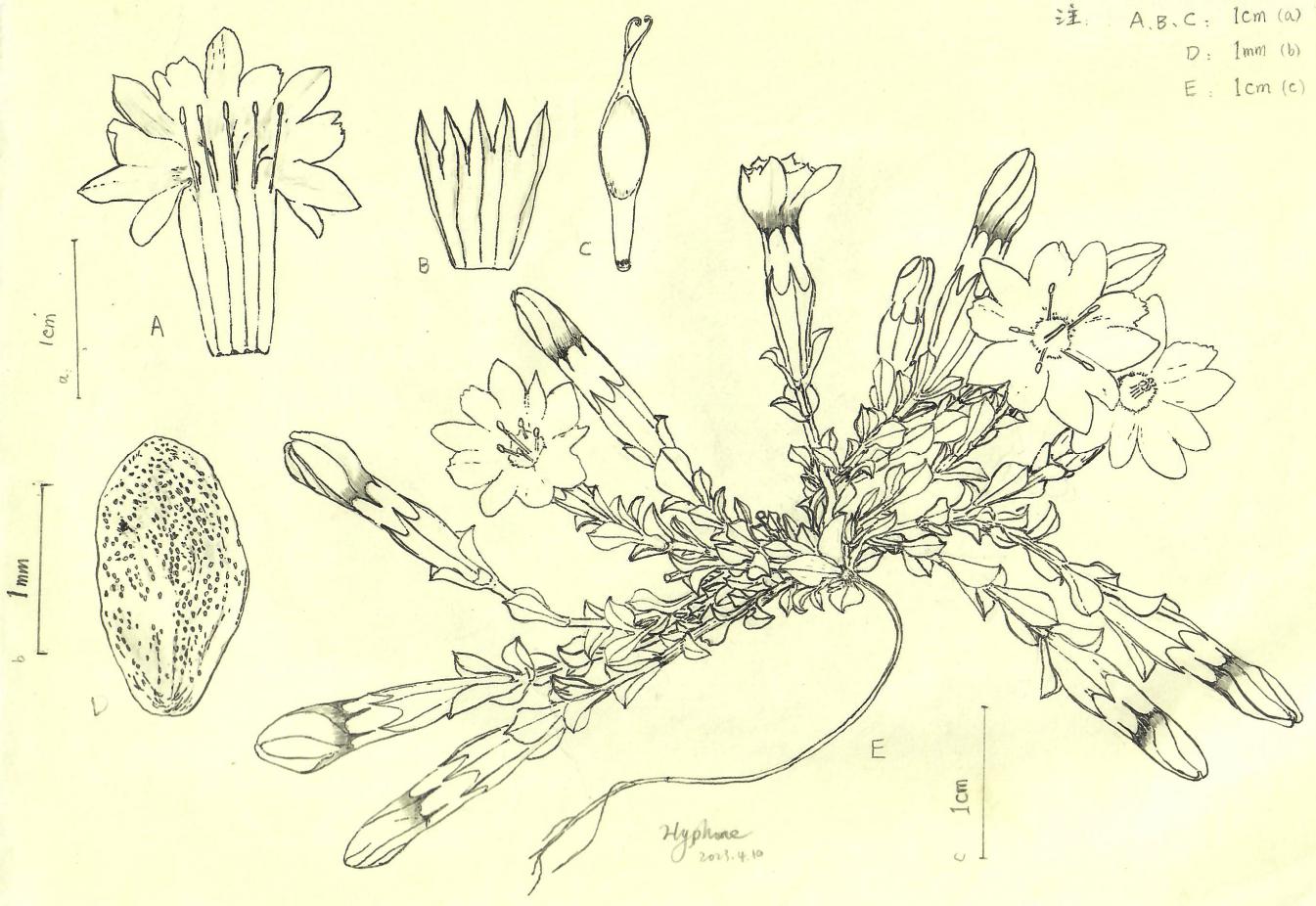


**Figure S5**. *Gentiana shangwui* H.F.Cao & L.Zhang. **A** Longitudinally opened corolla; **B** Longitudinally opened calyx; **C** Ovary & pistil; **D** Seed; **E** Habit. Scale bars: 1 cm (a) (**A, B, C**); 1 mm (b) (**D**); 1 cm (c) (**E**). **A–E** Drawn by H.F.Cao. **A–E** *L.Zhang 2018ZL0727*.
